# A Longitudinal Analysis Comparing the Mental Health of Children By Level of Young Carer Status

**DOI:** 10.1002/jad.12448

**Published:** 2024-12-16

**Authors:** Ed Janes, G. J. Melendez‐Torres

**Affiliations:** ^1^ CASCADE (Children's Social Care Research and Development Centre), School of Social Sciences Cardiff University Cardiff UK; ^2^ DECIPHer (Centre for Development, Evaluation, Complexity and Implementation in Public Health Improvement), School of Social Sciences Cardiff University Cardiff UK

**Keywords:** cross‐sectional, longitudinal, mental health, prevalence, young carers

## Abstract

**Introduction:**

Young carers research has predominantly focused on the experiences of children who often provide substantial levels of care for family members, and the impacts of this caring on their lives. While quantitative studies of prevalence have increased, there have been increasing calls for cross‐sectional and longitudinal studies of young carers relative to children without caring responsibilities, to strengthen and challenge the existing evidence on impact.

**Methods/Materials:**

The study utilized the Longitudinal Study of Young People in England: Next Steps (LSYPE), a cohort study of over 12,500 children aged 13 in 2004. The data set enabled the cross‐sectional and longitudinal study of young carers mental health relative to those without caring responsibilities. A descriptive analysis produced separate prevalence estimates for the whole young carer spectrum and those with more substantial responsibilities, and assessed caring impact on individual mental health aspects. This was a precursor to the structural equation modelling (SEM) of their overall mental health.

**Results:**

The findings highlighted the marginal or positive impacts of short‐term caring responsibilities, but also how mental health deteriorated over time. Both the short‐term benefits and long‐term deterioration of mental health was of a higher magnitude for those with more substantial responsibilities.

**Conclusion:**

The analysis of the larger young carer spectrum highlighted a diversity of positive and negative outcomes. While this was partly due to the size of the caring roles, duration of time in the carer role was a key factor in problematic caring roles.

## 1 Introduction

Young carer research, predominantly qualitative in method and conducted with those who access young carer projects, has explored the lives of children who provide care and support for family members. The evidence suggests that the life balance of education and social opportunities experienced by most children is affected by the caring role which, depending on the amount of responsibilities and the appropriateness of the caring tasks, can result in impacts on mental and physical health and wellbeing. At the same time, issues of identification have limited investigation of the larger population.

This article reports on the quantitative component of the *Caring Lives study* (Janes 2022) study that set out to explore the wider young carer spectrum and why the impacts of caring vary for children depending on their individual caring and family circumstances. The study used a broad definition of young carers as children under the age of 18 who have some level of caring responsibilities for a family member due to an illness or disability (including substance misuse or mental health issues). The results of a cross‐sectional and longitudinal study comparing (i) the whole young carer population, ii) those with more substantial responsibilities, and (iii) children without caring responsibilities are presented. Initial descriptive analysis of prevalence and individual mental health indicators is followed by the structural equation modelling (SEM) of overall mental health over time.

### 1.1 Progress in Quantitative Research

A recent narrative review of future directions in young carers research (Joseph et al. 2020) highlighted the need to recognise young carers as a large and diverse population, and argued for theoretically driven approaches with greater methodological rigor that seek to prove causation between caring and impacts. Large‐scale quantitative studies of young carers relative to children without caring responsibilities is seen as well‐suited to this task, but early research was hampered by a lack of large‐scale data.

### 1.2 Prevalence Studies

In the absence of suitable data, alternative methods were initially used to estimate young carer prevalence, as reviewed by Becker, Aldridge and Dearden (1998). This led to considerable variations and doubts over the true prevalence, but the increasing collection of data through confidential studies of large samples of children has led to a widely accepted range of 2−8% (Leu and Becker 2017), though higher estimates include 12% (Lloyd 2013) and 16% (Hewitt et al. 2019).

Part of this variation is likely due to the increasingly global scope of research, with the true prevalence likely to vary in different countries. However, Aldridge (2018) also highlighted variation in methods and issues with some studies including 18−25 year olds despite them increasingly being seen as a separate group of young adult carers, the lack of a young carer screening question, the child not being the respondent, and the poor application of instruments. Lastly, Aldridge noted variation in prevalence between studies that focus on young carers with substantial responsibilities to inform specialist services, and research into the whole population that seeks to understand when and why caring becomes problematic. If we accept that both research approaches are able to improve our understanding of young carers, there is value in both figures but also a necessity for clarity when reporting.

The majority of recent prevalence studies have reported on questions that did not define responsibilities as substantial or of a higher level. The resulting estimates therefore reflect the whole population of young carers (under 18) and include 7.9% in the United Kingdom (Nakanishi et al. 2022), 12% in Scotland (Robison, Inglis, and Egan 2020) and Northern Ireland (Lloyd 2013), 13.9% in England (Sharpe et al. 2021) and 16% in Wales (Hewitt et al. 2019). One study explicitly asked respondents if they cared for someone “seriously affected” by an illness or disability, resulting in a prevalence of 9.1% (Hamilton and Redmond 2020).

Five studies analysed an additional question on the amount of caring responsibilities to produce a prevalence of those with substantial responsibilities. Prevalence for substantial responsibilities was estimated in Austria and France at 4.5% and 12.25% respectively (Nagl‐Cupal et al. 2014; Pilato, Dorard, and Untas 2024), with no estimate of the wider population included. Leu et al. (2019) produced an estimate of 7.9% for the larger population in Switzerland, with almost 40% of them producing a high or very high level of care, and Meireles et al. (2023) used a similar method in Portugal, resulting in an overall prevalence of 10.6% and 67.9% of that group providing high or very high levels of care. De Roos, van Tienen, and de Boer (2020), cited in Lewis et al. (2022) produced an overall prevalence of 6−8% in the Netherlands, and a second prevalence of 3% providing intensive care (over 4 h each week). There is clear variation in estimates and these dual figures are vital in understanding the prevalence of substantial care in the context of the larger group.

### 1.3 Cross‐Sectional and Longitudinal Studies of Impact

Joseph et al. (2020) highlighted a lack of cross‐sectional or longitudinal studies of impact, limiting the ability to understand the impacts of caring by comparing young carers to children without caring responsibilities, or by considering change in outcomes over time. However, a recent systematic review (Fleitas Alfonzo et al. 2022) suggests that quantitative studies are becoming more complex, with almost all of the 10 included studies indicating poor mental health including greater depression and anxiety amongst carers compared to the general population. Other cross‐sectional studies have highlighted poor quality of life (Pilato, Dorard, and Untas 2024), mental health and wellbeing of young carers compared to peers (Leu et al. 2019; Lloyd 2013; Meireles et al. 2023; Nagl‐Cupal et al. 2014; Robison, Inglis, and Egan 2020; Sharpe et al. 2021). Nagl‐Cupal and Robison also found detrimental impacts on physical health, school and aspiration, and Vizard, Obolenskaya and Burchardt (2019) evidenced an increased susceptibility to poverty.

Comparison within the young carer population is rarer but, in addition to highlighting the lower wellbeing of adolescent carers compared to non‐carers, Lewis et al. (2022) found that these negative impacts increased in magnitude as caring responsibilities increased. Hamilton and Redmond (2020) found little difference in school engagement between young carers and other children, with the exception of reduced engagement amongst those caring for people with mental health and substance misuse issues.

Longitudinal studies have the benefit of better capturing the temporal relationship between young carer status and impacts. These are rare, but King, Singh and Disney (2021) evidenced that young carer status at age 14 was a predictor for poorer mental health at age 18‐19. Nakanishi et al. (2022) analysed data from the Millennium Cohort Study before and during the Covid‐19 pandemic and found young carer's mental health to be poor compared to those without caring responsibilities. However, these studies do not reflect the fact that young carer status can change over time, with some children having caring responsibilities from a young age while others transition into the role when they are deemed old enough or upon onset of a family illness. Equally, they can transition out of the role due to the death or recovery of the care receiver, or if other family members take on these responsibilities.

This study sought to use descriptive analysis and longitudinal SEM (structural equation modelling) to compare the mental health of three groups over time: young carers with substantial responsibilities; all children on the young carer spectrum; and children without caring responsibilities. With the exception of a study of benefit‐finding among young carers and young adult carers (Wepf, Joseph, and Leu 2021), and research into the effect of caring responsibilities on depressions (Kavanaugh 2013), SEM has not been used in young carers research.

## 2 Materials and Methods

### 2.1 Participants

Multiple cohort studies were assessed for the inclusion of key variables on young carer status and multiple aspects of mental health. The Longitudinal Study of Young People in England (LSYPE): Next Steps (University College London, UCL 2020), was selected as it was the only study to have longitudinal data for these variables, though there was variation in the data collected at each wave. Additional data concerned the amount of time spent caring, and demographic information concerning ethnicity, age and gender. Data was initially collected in 2004 from a cohort of 15,570 13‐year olds. Subsequent data were collected annually with 11,449 respondents at Wave Four, the latest data used in this study.

### 2.2 Procedure

This study used SEM, a versatile method suited to the cross‐sectional and longitudinal analysis of data. The approach is particularly useful for the analysis of multiple observable indicators that together represent an unobservable variable such as mental health (Kline 2016), and the modelling simultaneously analyses multiple hypothesised relationships. The procedure followed Kline's steps of specification, identification, estimation and reporting.

**Figure 1:**
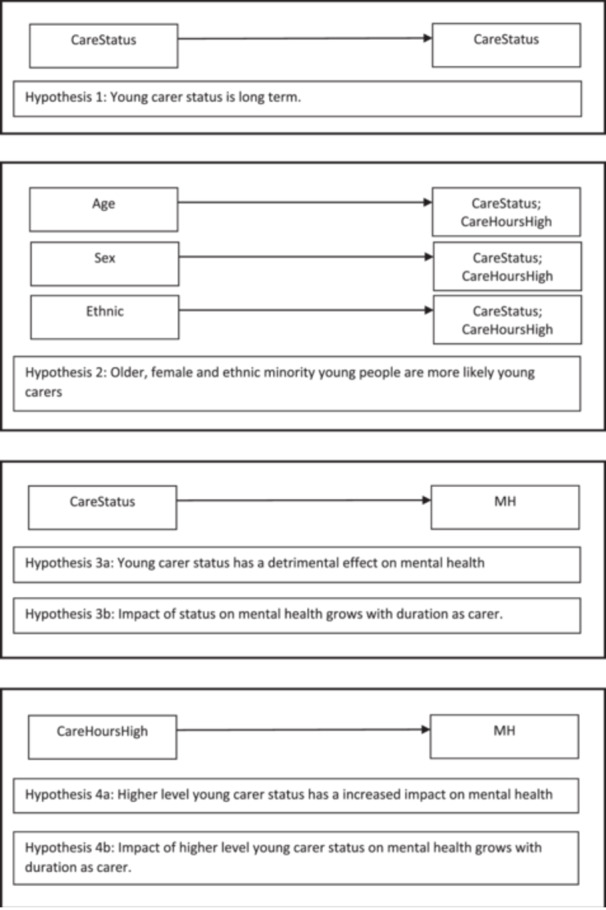
The hypotheses tested in the study were sourced from a realist review of why the impacts of caring varied for children depending on their caring and family circumstances (Janes et al. 2022).

The specification of six hypotheses (Figure 1) were theoretically informed by a realist review and model of how the impacts of caring varied for children depending on their family and caring circumstances (Janes et al. 2022). The first hypothesis states that young carer responsibilities are long‐term, while the second concerns prevalence being greater amongst older, female and ethnic minority children. Hypothesis Three states that young carer status is detrimental to mental health, with impacts expected to increase with duration in the caring role (Hypothesis Four). The final two hypotheses are similar but concern those with more substantial responsibilities, with increased time spent caring having a greater impact on mental health (Hypothesis Five), and these impacts again growing as the duration increases (Hypothesis Six).

### 2.3 Measures

#### 2.3.1 Young Carer Status

Table 1 displays the variable names, question wording, specific waves each variable was included in and response options. Wave One, Two and Three of the LSYPE cohort study included a status variable (*CareStatus*) with the following question: *Some people your age may have to look after other people. This could be a brother or sister, a relative or someone else who is disabled or sick. Is there anyone like this who lives here with you that you have to look after on a regular basis?* The binary variable (Yes = 1; No = 2) was suitable as it aligned with the definition in the study of young carers as a spectrum of children with different levels of caring responsibilities for any family member due to an illness or disability.

**Table 1:** Variables included in the models related to young carers, mental health and demographics.

Variable	Question	Wave	Scale
CareStatus	Some people your age may have to look after other people. This could be a brother or sister, a relative or someone else who is disabled or sick. Is there anyone like this who lives here with you that you have to look after on a regular basis?	1, 2, 3	1 = Yes, in this household; 2 = No
CareStatus1	Do you regularly look after any ill, disabled or elderly relatives or friends aged 15 or more and in need of care, without being paid? This includes both people who live here with you and those who live elsewhere	4, 5, 6, 7	1 = Yes; 2 = No
CareHours	About how many hours a week would you say that you usually spend looking after this person (these people) or doing things for them?	1, 2, 3	1 = 1−5; 2 = 6−10; 3 = 11−15; 4 = 16−20; 5 = 21+
SleepLoss	Have you recently lost much sleep over worry?	2, 4	1—Better than usual; 2—Same as usual;3—Less than usual;4—Much less than usual
UnderStrain	Have you recently felt constantly under strain?		
Difficulties	Have you recently felt you couldn't overcome your difficulties?		
EnjoyActivities	Have you recently been able to enjoy your normal day‐to‐day activities?		
FaceProblems	Have you recently been able to face up to your problems?		
Depressed	Have you recently been feeling unhappy and depressed?		
LowConfidence	Have you recently been losing confidence in yourself?		
Happy	Have you recently been feeling reasonably happy, all things considered?		
Concentration	Have you recently been able to concentrate on whatever you're doing?		
Useful	Have you recently felt you were playing a useful part in things?		
Decisive	Have you recently felt capable of making decisions about things?		
Worthless	Have you recently been thinking of yourself as a worthless person?		
Sex	Respondent is…:	1, 2, 3, 4, 5, 6, 7	1 = Male; 2 = Female
Ethnic	To which of the groups on this card would you say you belong?	1, 2, 4	1 = White; 2 = Mixed; 3 = Indian; 4 = Pakistani; 5 = Bangladeshi; 6 = Black Caribbean; 7 = Black African; 8 = Other

*Note:* CareStatus1 was not used due to change in meaning from previous CareStatus variable.

Wave Four onwards included a modified question as follows: *Do you regularly look after any ill, disabled or elderly relatives or friends aged 15 or more and in need of care, without being paid? This includes both people who live here with you and those who live elsewhere*. These changes were significant with the care receiver specified as over the age of 15, potentially discounting children who care for disabled siblings.

#### 2.3.2 Time Spent Caring

Those who identified as young carers were asked a supplementary question (*CareHours*) on the amount of time spent caring, enabling differentiation between the whole young carer population, and a smaller group with more substantial responsibilities. This was a continuous variable for Waves One and Two, and categorical for Wave Three (1 = 1−5; 2 = 6−10; 3 = 11−15; 4 = 16−20; 5 = 21+ hours).

#### 2.3.3 Mental Health

Waves Two, Four, and Seven included 12 possible variables for aspects of mental health (sleep loss due to worry; under strain; overcoming difficulties; enjoying day‐to‐day activities; facing problems; depressed; confidence; happy; concentration; feeling useful; making decisions; and worthlessness). Each variable was a 4‐point scale (1 = Better than usual; 2 = Same as usual; 3 = Less that usual; 4 = Much less than usual).

#### 2.3.4 Demographics

Waves One, Two, Three and Four included a binary *Sex* variable (1 = Male; 2 = Female). Waves, One, Two and Four included an ethnicity variable with eight options (1 = White; 2 = Mixed; 3 = Indian; 4 = Pakistani; 5 = Bangladeshi; 6 = Black Caribbean; 7 = Black African; 8 = Other). Inclusion of a variable for age was not necessary due to the longitudinal study collecting initial data when respondents were 13, and then at subsequent annual intervals.

### 2.4 Analysis

#### 2.4.1 Data Screening

The revised young carer status variable (*CareStatus*) for Wave Four onwards was not included in the analysis due to the significant change in the wording of the question. This altered the meaning of the question, resulting in the potential for the same respondent to answer the question differently despite having unchanged circumstances. The Wave Four variable for time spent caring (*CareHoursHigh)* was also excluded. Due to the young carer status variables only being available to Wave Three, the Wave Seven mental health variable was also discarded. Therefore the analysed data included young carer variables for Waves One, Two and Three, and m dental health variables for Waves Two and Four.

With the Wave Three time spent caring variable (*W3CareHours*) released as categorical data, the continuous Wave One and Two variables were recoded on the same scale. A new variable (*CareHoursHigh*) was then created to differentiate those with substantial roles (> 11 h per week), in line with the Young Carers – Making A Start study (Department of Health 1996). The four waves of the *Sex* variable were merged into a new *SexMerge* variable to reduce non‐responses, with similar merging of the three *Ethnic* variables into *EthnicMerge*.

The data was screened for univariate normality to identify potential irregularities, and multivariate normality to assess linear relationships between variables. Collinearity tests were conducted to check whether the mental health indicators for the two waves represented different facets of the same factor (*MH2* and *MH4* respectively), and this informed the decision to remove indicators from the analysis. A Principal Component Analysis (PCA) enabled the assessment of the optimal number of factors to explain the observed mental health indicator correlations. A confirmatory factor analysis (CFA) was then conducted to check the relationship strengths between each indicator and the factor that it loaded on, as well as the relationship strengths and the communality between the individual mental health indicators.

#### 2.4.2 SEM

Two models were identified. The main model used the *CareStatus* indicator to compare young carers with children without caring responsibilities. The additional model was identical except for the use of the *CareHoursHigh* indicator that enabled the comparison of young carers with substantial responsibilities and all other respondents.

Measurement invariance was assumed for the measurement model component, setting intercepts and thresholds for indicators equal across waves. Concerning the measurement component, the first mental health indicator, sleep loss, was a referent indicator and fixed to 1 for each factor ([*MH2* → *W2SleepLoss*] = [*MH4* →*W4SleepLoss*] = 1), with the remaining indicators calculated relative to this. Equality constraints were applied to the MH4 factor parameters, aligning them with the corresponding MH2 parameters (e.g., [*MH2* → *W2UnderStrain*] = [*MH4* → *W4UnderStrain*] = a2). All residual variances for the mental health factors and individual indicators were designated as free parameters to be estimated by the software. The categorical mental health indicators also needed thresholds: those loading onto the MH2 factor were designated free parameters, with equality constraints placed on the equivalent MH4 indicators (e.g., *e.W2SleepLoss* = *e.W4SleepLoss* = *b1*).

Concerning the structural components of the two models, all direct paths between demographic variables and the *CareStatus* variables (or *CareHoursHigh* variables in the additional model) were designated as free. All residual variances and intercepts for the respective young carer variables were also designated as free.

Models were checked for identification before estimation. Each model included 21 observed variables, a total of 50 free parameters and 202 degrees of freedom. With > 0 degrees of freedom required, estimation was therefore possible.

The models were estimated using MPlus version 8.3 (Muthén and Muthén 2017), specialist software developed for the SEM of categorical data. The MLR (Maximum Likelihood with Robust standard errors and a chi‐square test statistic) estimator enabled the incorporation of the categorical outcome variables into models, while the Monte Carlo algorithm was specified for the purpose of modelling data with missing values.

Estimation of both models completed successfully and without the need for respecification. The models are therefore a priori and fully theoretically informed by the young carer realist review (Janes et al. 2022). The scripts for the estimation of the models are included in the supplementary information (SM1).

## 3 Results

### 3.1 Data Screening Results

**Figure 2:**
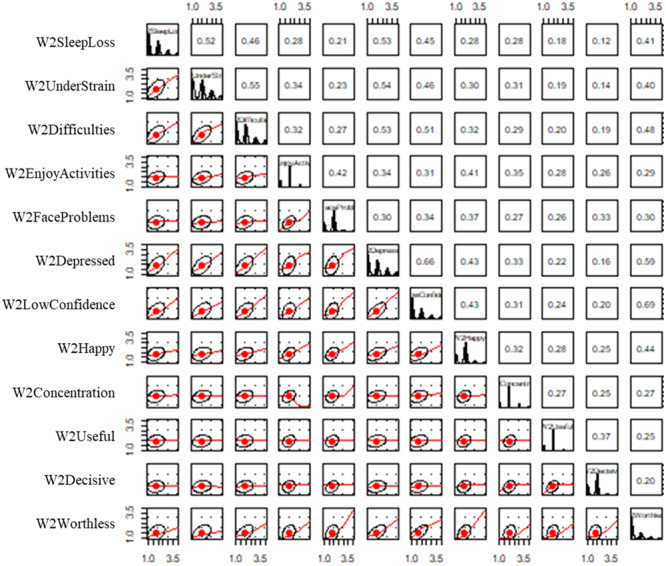
Scatter plot matrices for linear relationships between MH2 mental health indicators.

**Figure 3:**
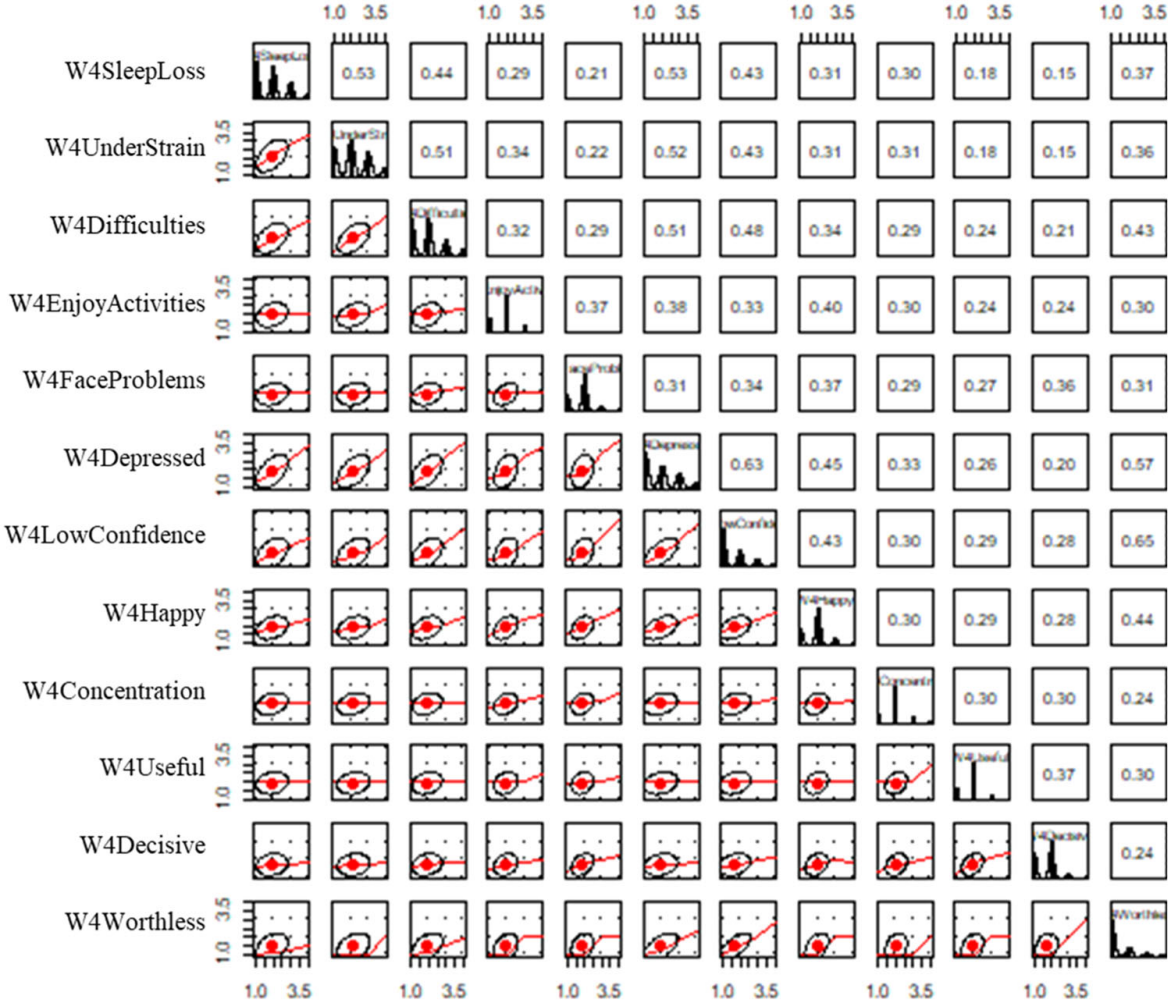
Scatter plot matrices for linear relationships between MH4 mental health indicators.

Scatterplot matrices highlighted a poor linear relationship between worthlessness and the other mental health indicators (Figures 2 and 3). The correlation matrix (Table 2) also identified low correlations (< 0.30) between multiple pairs of indicators, suggesting that they did not represent the same factors. In particular, the indicators for worthlessness, usefulness, decision making and concentration had repeated collinearity with other indicators, resulting in these indicator being removed. Each mental health factor was therefore represented by eight indicators including sleep loss due to worry, strain, overcoming difficulties, enjoying activities, facing problems, depressed, confidence and happiness.

**Table 2:** Correlation matrices for mental health indicators, with Wave Two indicators below the diagonal and Wave Four indicators above.

	W4 SleepLoss	W4 UnderStrain	W4 Difficulties	W4 EnjoyActivities	W4 FaceProblems	W4 Depressed	W4 LowConfidence	W4 Happy	W4 Concentrate	W4 Useful	W4 Decisive	W4 Worthless
W2SleepLoss		0.53	0.44	0.29	0.21	0.53	0.43	0.31	0.30	0.18	0.15	0.37
W2UnderStrain	0.52		0.51	0.34	0.22	0.52	0.43	0.31	0.31	0.18	0.15	0.36
W2Difficulties	0.46	0.55		0.32	0.29	0.51	0.48	0.34	0.29	0.24	0.21	0.43
W2EnjoyActivities	0.28	0.34	0.32		0.37	0.38	0.33	0.40	0.30	0.24	0.24	0.30
W2FaceProblems	0.21	0.23	0.27	0.42		0.31	0.34	0.37	0.29	0.27	0.36	0.31
W2Depressed	0.53	0.54	0.53	0.34	0.30		0.63	0.45	0.33	0.26	0.20	0.57
W2LowConfidence	0.45	0.46	0.51	0.31	0.34	0.66		0.43	0.30	0.29	0.28	0.65
W2Happy	0.28	0.30	0.32	0.41	0.37	0.43	0.43		0.30	0.29	0.28	0.44
W2Concentrate	0.28	0.31	0.29	0.35	0.27	0.33	0.31	0.32		0.30	0.30	0.24
W2Useful	0.18	0.19	0.20	0.28	0.26	0.22	0.24	0.28	0.27		0.37	0.30
W2Decisive	0.12	0.14	0.19	0.26	0.33	0.16	0.20	0.25	0.25	0.37		0.24
W2Worthless	0.41	0.40	0.48	0.29	0.30	0.59	0.69	0.44	0.27	0.25	0.20	

*Note:* The four bottom indicators (concentration; usefulness; decision making; worthlessness) were excluded in the data screening stage.

The PCA indicated the need for a single mental health factor. The results of this and the Maximum Likelihood CFA are reported in the supplementary information (SM2).

**Figure 4:**
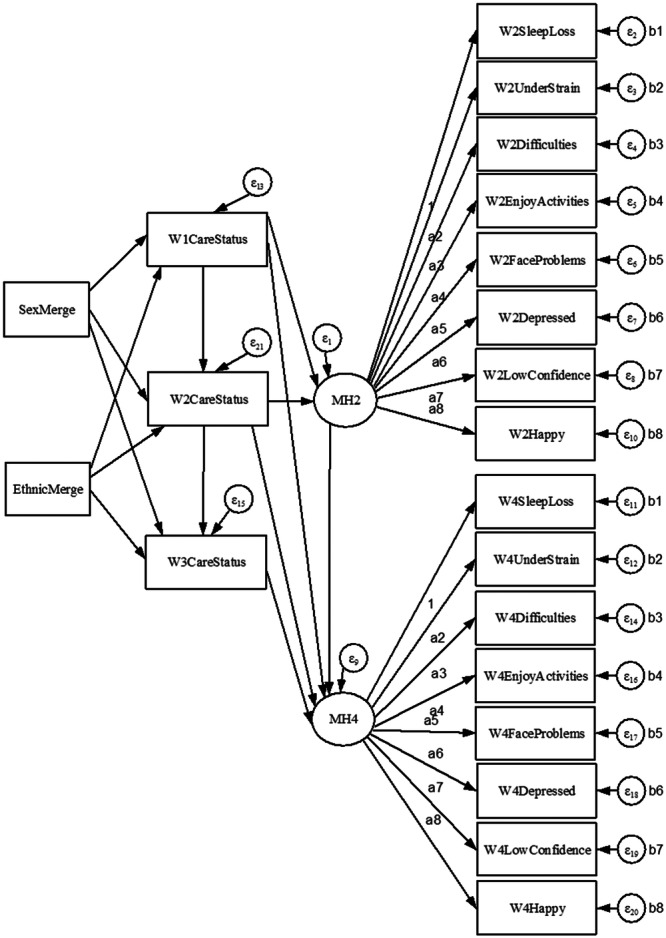
Identified main model for comparing the mental health of young carers and children without caring responsibilities over time.

**Figure 5:**
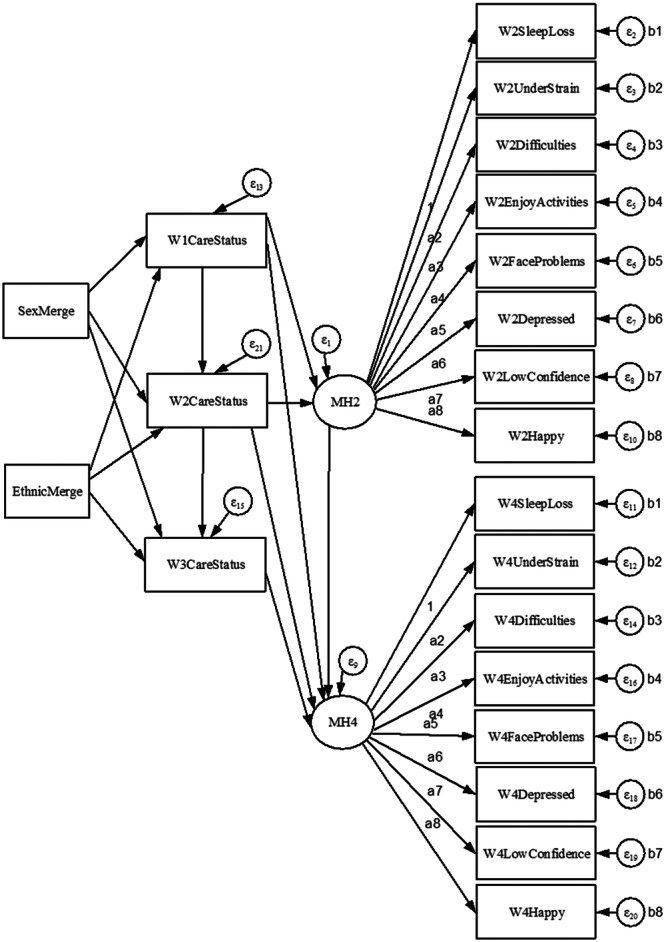
Identified additional model for comparing the mental health of those with substantial responsibilities to other respondents over time.

The young carer status model is displayed in Figure 4, with the additional higher‐level responsibilities model in Figure 5.

### 3.2 Descriptive Analysis

#### 3.2.1 Prevalence

At all three timepoints, the subset of respondents who identified as young carers was over 750 (Table 3). Young carer prevalence increased with age, from 5.13% at age 13 to 5.76% and 6.21% at age 14 and 15 respectively. Prevalence at all three timepoints was greater for females (5.40%; 6.23%; 6.64%) than males (4.87%; 5.30%; 5.78%), and for minority ethnic children (6.99%; 7.93%; 7.92%) compared to white children (4.22%; 4.75%; 5.44%).

**Table 3:** Prevalence results for all young carers (left) and those with substantial responsibilities (right), including by age, sex and ethnic demographics.

	Carer prevalence					Higher level carer prevalence				
Data wave	Demographic	*N*	Carer	Non carer	Prevalence	Demographic	*N*	High carer	Standard/non carer	Prevalence
	Age					Age				
W1	13	15,411	791	14,620	5.13%	13	15,345	121	15,224	0.79%
W2	14	13,273	764	12,509	5.76%	14	13,213	114	13,099	0.86%
W3	15	12,276	762	11,514	6.21%	15	12,236	126	12,110	1.03%
	Gender					Gender				
W1	Male	7837	382	7455	4.87%	Male	7810	51	7759	0.65%
	Female	7574	409	7165	5.40%	Female	7535	70	7465	0.93%
W2	Male	6721	356	6365	5.30%	Male	6686	48	6638	0.72%
	Female	6552	408	6144	6.23%	Female	6527	66	6461	1.01%
W3	Male	6192	358	5834	5.78%	Male	6178	48	6130	0.78%
	Female	6084	404	5680	6.64%	Female	6058	78	5980	1.29%
	Ethnicity					Ethnicity				
W1	White	10329	436	9893	4.22%	White	10,300	56	10,244	0.54%
	ME	5080	355	4725	6.99%	ME	5043	65	4978	1.29%
W2	White	9073	431	8642	4.75%	White	9034	53	8981	0.59%
	ME	4199	333	3866	7.93%	ME	4178	61	4117	1.46%
W3	White	8474	461	8013	5.44%	White	8453	62	8391	0.73%
	ME	3800	301	3499	7.92%	ME	3781	64	3717	1.69%

The subset of young carers with substantial responsibilities was limited to under 130 respondents in each wave, resulted in the data being interpreted with caution. Prevalence for this group again increased over the 3 years, from 0.79% to 0.86% and 1.03%. Similarly to the young carer spectrum prevalence estimates, those with more substantial responsibilities were more likely female at each timepoint (0.93%; 1.01%; 1.29%) than male (0.65%; 0.72%; 0.78%), and from ethnic minority populations (1.29%; 1.46%; 1.69%) than white (0.54%; 0.59%; 0.73%).

#### 3.2.2 Mental Health

Table 4 displays the comparative mental health of all young carers versus children without caring responsibilities, and those with substantial responsibilities versus other respondents. Considering the larger subset first, young carers scored marginally higher on each of the eight Wave Two mental health indicators, indicating poorer outcomes. This was most notable and statistically significant for depression (1.97 compared to 1.87, *df* = 801.41; *t* = −2.55; *p* = 0.011). The results were similar at Wave Four, with the exception of young carers being better able to face problems though this finding was not statistically significant (1.83 compared to 1.87; *df* = 747.17; *t* = 1.40; *p* = 0.163). With young carer status changing over time, the lack of a stable sample prevented the use of paired t‐tests to assess the statistical significance of any longitudinal change, but higher scores on six of the eight variables at Wave Four indicated a deterioration in mental health, especially concerning strain (+0.17) and the enjoyment of daily activities (+0.11). However, this coincided with worsening mental health amongst non‐young carers.

**Table 4:** Descriptive statistics comparing young carers to children without caring responsibilities for eight individual mental health variables.

		Carer			Non carer			*T* tests		
Wave	Indicator	*N*	Mean	SD	*N*	Mean	SD	Dof	*T* value	*p* value
2	W2SleepLoss	717	1.89	0.94	11,970	1.77	0.88	793.85	−3.47	0.000
4	W4SleepLoss	670	1.97	0.95	10,271	1.92	0.91	752.11	−1.44	0.151
2	W2UnderStrain	683	1.98	0.94	11,557	1.96	0.94	764.96	−0.42	0.678
4	W4UnderStrain	666	2.15	0.96	10,235	2.14	0.94	750.72	−0.18	0.854
2	W2Difficulties	682	1.89	0.93	11,520	1.85	0.89	756.8	−1.07	0.285
4	W4Difficulties	659	1.92	0.92	10,229	1.88	0.88	736.42	−1.04	0.300
2	W2EnjoyActivities	716	1.9	0.72	12,001	1.9	0.64	783.92	−0.16	0.871
4	W4EnjoyActivities	675	2.01	0.75	10,321	1.95	0.7	752.4	−2.04	0.042
2	W2FaceProblems	707	1.84	0.74	11,955	1.83	0.64	769.71	−0.47	0.636
4	W4 FaceProblems	673	1.83	0.7	10,289	1.87	0.64	747.17	1.40	0.163
2	W2Depressed	718	1.97	0.99	11,868	1.87	0.96	801.41	−2.55	0.011
4	W4Depressed	663	2.03	1.05	10,230	1.9	0.97	738.44	−3.29	0.001
2	W2LowConfidence	722	1.78	0.99	11,945	1.7	0.9	796.17	−2.22	0.268
4	W4LowConfidence	666	1.76	0.98	10,273	1.69	0.9	739.34	−1.77	0.077
2	W2Happy	691	1.91	0.76	11,627	1.87	0.68	757.5	−1.61	0.108
4	W4Happy	663	1.94	0.72	10,229	1.9	0.67	739.99	−1.34	0.182

*Note:* Young carer sample was not constant due to status potentially changing over time. Therefore, the use of paired *t*‐tests to assess the statistical significance of longitudinal change was not possible.

For those with substantial responsibilities, their mental health at Wave Two was better for seven of the eight indicators (Table 5), indicating better mental health than other respondents. Scores were particularly low for the facing of problems (1.71 compared to 1.83; *df* = 102.61; *t* = 1.85; *p* = 0.067), while the exception was sleep loss (1.83 compared to 1.77; *df* = 109.71; *t* = −0.61; *p* = 0.544). However, the short‐term mental health benefits were reversed at Wave Four, with the subset having higher scores on six indicators. In particular, their ability to enjoy activities (2.12 compared to 1.95; *df* = 109.47; *t* = −2.07; *p* = 0.040), and their quality of sleep (2.05 compared to 1.92; *df* = 110.63; *t* = −1.23; *p* = 0.221) was poor. The mental health of those with greater responsibilities therefore deteriorated longitudinally, especially concerning activity enjoyment (+0.33), strain (+0.25) and sleep loss (+0.22), though this trend of deteriorating mental health was again apparent in the wider population.

**Table 5:** Descriptive statistics comparing young carers with substantial responsibilities to other respondents for eight individual mental health variables.

		Higher level carer			Standard/non carer			*T* Tests		
Wave	Indicator	*N*	Mean	SD	*N*	Mean	SD	Dof	*T* value	*p* value
2	W2SleepLoss	109	1.83	0.93	12,527	1.77	0.89	109.71	−0.61	0.544
4	W4SleepLoss	110	2.05	1.07	10,794	1.92	0.91	110.63	−1.23	0.221
2	W2UnderStrain	93	1.88	0.92	12,098	1.97	0.94	93.498	0.88	0.38
4	W4UnderStrain	106	2.13	1.01	10,760	2.14	0.95	106.8	0.07	0.943
2	W2Difficulties	97	1.82	0.97	12,057	1.85	0.89	97.313	0.26	0.798
4	W4Difficulties	106	1.88	0.92	10,748	1.88	0.88	106.88	0.07	0.944
2	W2EnjoyActivities	105	1.79	0.69	12,560	1.9	0.64	105.53	1.60	0.113
4	W4EnjoyActivities	109	2.12	0.85	10,851	1.95	0.7	109.47	−2.07	0.040
2	W2FaceProblems	102	1.71	0.65	12,509	1.83	0.65	102.61	1.85	0.067
4	W4 FaceProblems	110	1.91	0.84	10,815	1.86	0.64	110.29	−0.57	0.571
2	W2Depressed	105	1.88	0.94	12,431	1.88	0.96	105.86	0.00	0.997
4	W4Depressed	108	2.01	1.1	10,749	1.9	0.98	108.71	−1.01	0.316
2	W2LowConfidence	108	1.67	0.92	12,507	1.7	0.91	108.82	0.42	0.678
4	W4LowConfidence	110	1.74	1.04	10,793	1.69	0.91	110.7	−0.45	0.651
2	W2Happy	98	1.83	0.76	12,171	1.87	0.68	98.266	0.53	0.594
4	W4Happy	108	2.02	0.74	10,748	1.9	0.67	108.82	−1.67	0.097

*Note:* The subgroup of young carers with substantial responsibilities was not constant due to status potentially changing over time. Therefore, the use of paired *t*‐tests to assess the statistical significance of longitudinal change was not possible.

### 3.3 SEM Results

The descriptive analysis reported the dual prevalence rates, and compared all young carers, those with substantial responsibilities, and children without caring responsibilities for eight individual mental health indicators. While the results on how the impacts change over time are of interest, the SEM of the eight indicators as a single latent factor, and the simultaneous analysis of all the parameters enables the strengthening of these findings.

### 3.4 Estimation

Estimation of the main young carer model resulted in the exclusion of 199 cases due to insufficient data, with 15,923 cases included. Response rates for all variables was high (> 71%), particularly the young carer status variables (*W1CareStatus* = 96.8%; *W2CareStatus* = 83.4%; *W3CareStatus* = 77.1%). Covariance response was also high for all variable pairs (> 63%).

Considering the additional model on substantial responsibilities, missing data was slightly greater for the *CareHoursHigh* indicators, resulting in the exclusion of 216 cases. Despite this, 15906 cases were included, with response rates again high for all variables (> 71%) and the substantial care variables (*W1CareHoursHigh* = 96.5%; *W2CareHoursHigh* = 83.1%; *W3CareHoursHigh* = 76.9%). Covariance response also remained high (> 63%).

### 3.5 Measurement Component

**Figure 6:**
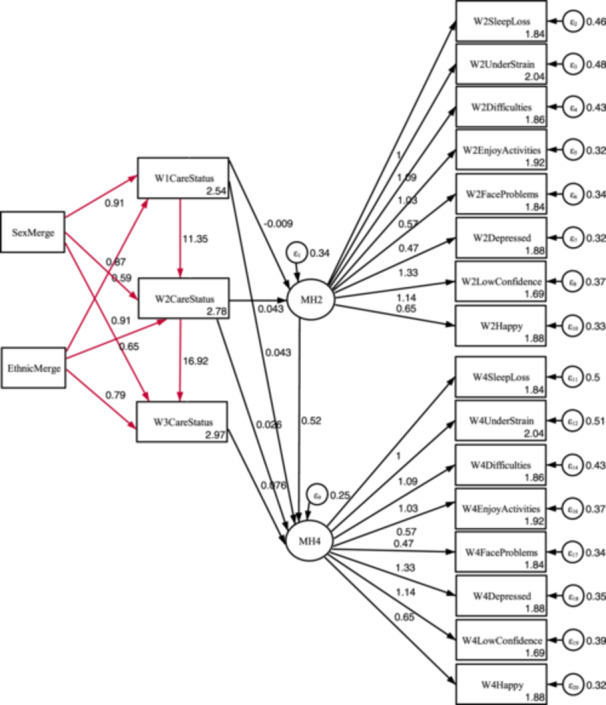
Path diagram for the main young carer status model. NB. Black paths = coefficients (*β*), Red paths = Odds ratios (OR).

**Figure 7:**
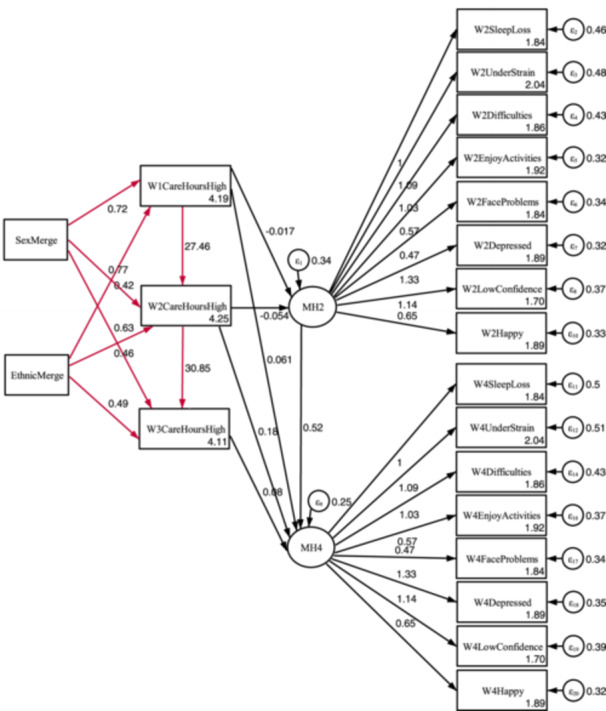
Path diagram for the additional substantial responsibilities model. NB. Black paths = coefficients (β), Red paths = Odds ratios (OR).

The tabulated results of the models are displayed in Table 6, and the separate path diagrams are displayed in Figures 6 and 7. With the only difference in the models being the use of the *CareStatus* or *CareHoursHigh* variable in the structural component of the model, the results of the measurement component are near identical with any differences due to the differing number of excluded cases. The results are therefore reported together.

**Table 6:** Parameter estimates for the main young carer status model (left) and the additional substantial responsibilities model (right).

Young carer model				Higher level young carer model			
Paths			Estimate		Paths		Estimate
Factor loading				Factor loading			
	MH2 BY				MH2 BY		
		W2SLEEPLOSS	1			W2SLEEPLOSS	1
		W2UNDERSTRAIN	1.092***			W2UNDERSTRAIN	1.092***
		W2DIFFICULTIES	1.026***			W2DIFFICULTIES	1.026***
		W2ENJOYACTIVITIES	0.567***			W2ENJOYACTIVITIES	0.567***
		W2FACEPROBLEMS	0.472***			W2FACEPROBLEMS	0.472***
		W2DEPRESSED	1.331***			W2DEPRESSED	1.331***
		W2LOWCONFIDENCE	1.144***			W2LOWCONFIDENCE	1.145***
		W2HAPPY	0.645***			W2HAPPY	0.645***
	MH4 BY				MH4 BY		
		W4SLEEPLOSS	1			W4SLEEPLOSS	1
		W4UNDERSTRAIN	1.092***			W4UNDERSTRAIN	1.092***
		W4DIFFICULTIES	1.026***			W4DIFFICULTIES	1.026***
		W4ENJOYACTIVITIES	0.567***			W4ENJOYACTIVITIES	0.567***
		W4FACEPROBLEMS	0.472***			W4FACEPROBLEMS	0.472***
		W4DEPRESSED	1.331***			W4DEPRESSED	1.331***
		W4LOWCONFIDENCE	1.144***			W4LOWCONFIDENCE	1.145***
		W4HAPPY	0.645***			W4HAPPY	0.645***
Residual variances				Residual variances			
		W2SLEEPLOSS	0.458***			W2SLEEPLOSS	0.458***
		W2UNDERSTRAIN	0.481***			W2UNDERSTRAIN	0.481***
		W2DIFFICULTIES	0.425***			W2DIFFICULTIES	0.425***
		W2ENJOYACTIVITIES	0.319***			W2ENJOYACTIVITIES	0.319***
		W2FACEPROBLEMS	0.343***			W2FACEPROBLEMS	0.343***
		W2DEPRESSED	0.324***			W2DEPRESSED	0.324***
		W2LOWCONFIDENCE	0.366***			W2LOWCONFIDENCE	0.366***
		W2HAPPY	0.333***			W2HAPPY	0.333***
		W4SLEEPLOSS	0.495***			W4SLEEPLOSS	0.496***
		W4UNDERSTRAIN	0.505***			W4UNDERSTRAIN	0.505***
		W4DIFFICULTIES	0.429***			W4DIFFICULTIES	0.429***
		W4ENJOYACTIVITIES	0.37***			W4ENJOYACTIVITIES	0.37***
		W4FACEPROBLEMS	0.342***			W4FACEPROBLEMS	0.342***
		W4DEPRESSED	0.347***			W4DEPRESSED	0.348***
		W4LOWCONFIDENCE	0.388***			W4LOWCONFIDENCE	0.388***
		W4HAPPY	0.316***			W4HAPPY	0.316***
		MH2	0.344***			MH2	0.344***
		MH4	0.251***			MH4	0.251***
Factor correlation				Factor correlation			
	MH4 ON				MH4 ON		
		MH2	0.521***			MH2	0.521***
Direct effects				Direct effects			
	MH2 ON				MH2 ON		
		W1CARESTATUS	−0.009			W1CAREHOURSHIGH	−0.017
		W2CARESTATUS	0.043			W2CAREHOURSHIGH	−0.054
	MH4 ON				MH4 ON		
		W1CARESTATUS	0.043			W1CAREHOURSHIGH	0.061
		W2CARESTATUS	0.026			W2CAREHOURSHIGH	0.175*
		W3CARESTATUS	0.076**			W3CAREHOURSHIGH	0.08
	W1CARESTATUS ON				W1CAREHOURSHIGH ON		
		SEXMERGE	−0.097			SEXMERGE	−0.326
		ETHNICMERG	−0.533***			ETHNICMERG	−0.863***
	W2CARESTATUS ON				W2CAREHOURSHIGH ON		
		W1CARESTATUS	2.429***			W1CAREHOURSHIGH	3.313***
		SEXMERGE	−0.136			SEXMERGE	−0.262
		ETHNICMERG	−0.431***			ETHNICMERG	−0.77***
	W3CARESTATUS ON				W3CAREHOURSHIGH ON		
		W2CARESTATUS	2.829***			W2CAREHOURSHIGH	3.429***
		SEXMERGE	−0.098			SEXMERGE	−0.462*
		ETHNICMERG	−0.231**			ETHNICMERG	−0.707***
Logistic regression odds ratio results				Logistic regression odds ratio results			
	W2CARESTATUS ON				W2CAREHOURSHIGH ON		
		W1CARESTATUS	11.348***			W1CAREHOURSHIGH	27.458**
		SEXMERGE	0.873			SEXMERGE	0.769
		ETHNICMERG	0.65***			ETHNICMERG	0.463***
	W3CARESTATUS ON				W3CAREHOURSHIGH ON		
		W2CARESTATUS	16.924***			W2CAREHOURSHIGH	30.845**
		SEXMERGE	0.907			SEXMERGE	0.63**
		ETHNICMERG	0.794**			ETHNICMERG	0.493***
	W1CARESTATUS ON				W1CAREHOURSHIGH ON		
		SEXMERGE	0.907			SEXMERGE	0.722*
		ETHNICMERG	0.587***			ETHNICMERG	0.422***
Intercepts				Intercepts			
		W2SLEEPLOSS	1.836***			W2SLEEPLOSS	1.841***
		W2UNDERSTRAIN	2.038***			W2UNDERSTRAIN	2.043***
		W2DIFFICULTIES	1.859***			W2DIFFICULTIES	1.864***
		W2ENJOYACTIVITIES	1.921***			W2ENJOYACTIVITIES	1.923***
		W2FACEPROBLEMS	1.84***			W2FACEPROBLEMS	1.842***
		W2DEPRESSED	1.882***			W2DEPRESSED	1.888***
		W2LOWCONFIDENCE	1.692***			W2LOWCONFIDENCE	1.698***
		W2HAPPY	1.883***			W2HAPPY	1.886***
		W4SLEEPLOSS	1.836***			W4SLEEPLOSS	1.841***
		W4UNDERSTRAIN	2.038***			W4UNDERSTRAIN	2.043***
		W4DIFFICULTIES	1.859***			W4DIFFICULTIES	1.864***
		W4ENJOYACTIVITIES	1.921***			W4ENJOYACTIVITIES	1.923***
		W4FACEPROBLEMS	1.84***			W4FACEPROBLEMS	1.842***
		W4DEPRESSED	1.882***			W4DEPRESSED	1.888***
		W4LOWCONFIDENCE	1.692***			W4LOWCONFIDENCE	1.698***
		W4HAPPY	1.883***			W4HAPPY	1.886***
Thresholds				Thresholds			
		W1CARESTATUS$1	2.541***			W1CAREHOURSHIGH$1	4.19***
		W2CARESTATUS$1	2.778***			W2CAREHOURSHIGH$1	4.345***
		W3CARESTATUS$1	2.965***			W3CAREHOURSHIGH$1	4.113***

*** *p* < 0.001.

** *p* < 0.01.

* *p* < 0.05.

With the exception of the designated referent indicator as a constrained parameter (*β* = 1), all loading of indicators on the mental health factors were statistically significant (*p* < 0.001). Factor loading was greatest for depression (*β* = 1.331), and lowest but still moderate for facing problems (*β* = 0.472). Residual variances within the measurement component were highest for strain (W2UnderStrain var = 0.481; W4UnderStrain var = 0.505) but still low to moderate and statistically significant (*p* < 0.001).

There was a statistically significant positive correlation between *MH2* and *MH4*, indicating that mental health at age 14 was a significant predicator for mental health at age 16. Residual variance was low with approximately a third unexplained for *MH2* (*β* = 0.344; *p* < 0.001), and lower at *MH4* (*β* = 0.251; *p* < 0.001).

### 3.6 Structural Component

The results of the structural component are considered in order of the six hypotheses. The sizeable and statistically significant odds ratios evidence that young carer status is long‐term (*CareStatus1 → CareStatus2*, OR = 11.348; *CareStatus2 → CareStatus3*, OR = 16.924). The equivalent results for the substantial care model were of a greater magnitude (*CareHoursHigh1 → CareHoursHigh2*, OR = 27.458; *CareHoursHigh2 → CareHoursHigh3*, OR = 30.845), indicating that those with greater responsibilities are also more likely to be long‐term carers.

Hypotheses Two concerned the prevalence rates for ethnicity and gender. Young carer spectrum prevalence was greater for ethnic minority children than white children at all timepoints (*EthnicMerge → W1CareStatus*, OR = 0.587; *EthnicMerge → W2CareStatus*, OR = 0.650; *EthnicMerge→ W3CareStatus*, OR = 0.794) with all results statistically significant (*p* < 0.01). This was of a greater magnitude amongst those with substantial responsibilities (*EthnicMerge → W1CareStatus*, OR = 0.422; EthnicMerge → *W2CareStatus*, OR = 0.463; EthnicMerge→*W3CareStatus*, OR = 0.493) and the findings were again significant (*p* < 0.001).

The evidence was weaker concerning difference in prevalence by sex. Females were more likely members of the young carer spectrum, but none of the three parameters were statistically significant (*SexMerge → W1CareStatus*, OR = 0.907, *p* = 0.164; *SexMerge → W2CareStatus*, OR = 0.873, *p* = 0.064; *SexMerge → W3CareStatus*, OR = 0.907, *p* = 0.205). The findings for the substantial carer model were of a greater magnitude and significant at Wave One and Three, with females more likely to have greater responsibilities (*SexMerge → W1CareHoursHigh*, OR = 0.722; *p* < 0.05; *SexMerge → W2CareHoursHigh*, OR = 0.769; *p* = 0.122; *W3CareStatus*, OR = 0.630; *p* < 0.01).

Hypotheses Three and Four concerned impact of young carer status on mental health, and whether this impact increased with duration in the role. The short‐term impacts were inconclusive and not statistically significant, with Wave One care status having a marginal beneficial impact on mental health (*W1CareStatus → MH2*, β = −0.009, *p* = 0.738), and Wave Two status a slight negative impact (*W2CareStatus → MH2*, *β* = −0.043, *p* = 0.103). However, care status at Waves One, Two and Three had long term negative impacts on mental health at Wave Four (*W1CarerStatus → MH4*, *β* = 0.043, *p* = 0.121; *W2CarerStatus → MH4*, *β* = 0.026, *p* = 0.339; *W3CarerStatus → MH4*, *β* = 0.076, *p* < 0.01), with statistical significance at Wave Three.

The final two hypotheses concern impact of substantial responsibilities on mental health, and how these impacts changed over time. The short‐term impacts of care status at Waves One and Two on Wave Two mental health were beneficial (*W1CareHoursHighs → MH2*, *β* = −0.017; *p* = 0.797; *W2CareHoursHigh → MH2*, *β* = −0.054, *p* = 0.389) though the findings were not statistically significant, potentially due to the small subsample of respondents. Hypothesis Six, that negative impacts increased with duration of the substantial responsibilities was also supported (*W1CareHoursHigh → MH4*, *β* = 0.061, *p* = 0.372; *W3CareHoursHigh → MH4; β* = 0.08, *p* = .282), with statistical significance at Wave Two.

## 4 Discussion

The review by Joseph et al. (2020) of potential future directions included the need to recognise the larger young carer population, and to use quantitative methods that strengthen past evidence by studying them relative to children without caring responsibilities. Furthermore, studies of the whole young carer population has at times found negative effects that are statistically significant but marginal (Lewis et al. 2022; Lloyd 2013; Meireles et al. 2023), suggesting the need to explore difference within the diverse population. This study has sought to investigate the whole population through the analysis of a large longitudinal cohort study of over 15,500 young people. The study includes a sample of over 750 young carers that compares favourably to other research, but also approximately 120 with more substantial responsibilities, enabling comparison between young carers and children without caring responsibilities, but also within the group.

Two sets of prevalence estimates were produced, with 5.1−6.2% of all respondents across the three waves of data having caring responsibilities for a family member due to an illness or disability, and 0.8−1.0% caring for over 11 h each week. The study also strengthens the evidence that the prevalence of young carers, as well as those with substantial roles, is greater amongst older, female and minority children.

While the initial descriptive analysis evidenced deterioration in individual mental health indicators amongst young carers over time, especially those with substantial responsibilities, this trend was also apparent amongst children without caring responsibilities. The use of SEM, and the development of a priori models fully informed by theory enabled the comparative analysis of the overall mental health of young carers relative to non‐young carers.

Previous research has considered substantial caring responsibilities in terms of excessive time spent caring or the undertaking of responsibilities deemed inappropriate for children. This study reinforces the findings in relation to amount of time spent caring being linked to impacts, but there has been less focus on importance of the duration of time in the young carer role. The results of the study suggest that duration is key, but also that the two factors interact, with evidence that those with substantial responsibilities have better short‐term mental health than both other young carers and children without caring responsibilities together, but that they have significantly poorer long‐term outcomes. This is in comparison to the whole young carer population where short‐terms impacts were marginal, and where mental health deteriorated but to a lesser extent. Further research into this is needed, but one possible explanation for the short‐term benefits is that those with substantial caring responsibilities more likely care for a seriously ill family member, and that being able to help acts as a short‐term protective factor which is lost over time.

### 4.1 Limitations and Opportunities for Future Studies

This study used cohort data from the first LSYPE (Next Steps) as it was the only data set at the time of analysis to include multiple waves of data on young carer status. The use of already collected data placed limitations on what could be studied. Specifically, the realist review underpinning the SEM models (Janes et al. 2022) included theory relating to inappropriate caring responsibilities, being known to services and the accessing of support, and data was not available relating to this. These variables are also rarely collected in other cohort studies, and there is a need to expand the data that is collected.

Considering the dual prevalence estimates of 5.1−6.2% for all young carers, and 0.8−1.0% for those with substantial responsibilities, these figures are lower than many recent studies. With the data collected between 2004 and 2007, this difference may be partly explained by a change in the true prevalence over time, but it is also possible that increased self‐awareness and reduced stigma has resulting in the increased accuracy of recent confidential cohort studies. Longitudinal analysis of more recent data is needed.

The decision to define those with substantial responsibilities as caring for over 11 h a week was partly due to the Wave Three time spent caring variable (*W3CareHours*) being a categorical indicator, though this did align with the Making a Start report (Department of Health 1996). While the size of the cohort study and the young carer subsample are both notably high, the number of respondents with substantial caring responsibilities is low, limiting the potential for statistically significant findings. Large‐scale quantitative studies of those with substantial responsibilities relative to other children are however challenging given the low prevalence.

The threshold for substantial caring responsibilities also varies in different studies, with examples including time spent caring totalling over 4 h per week (de Roos, van Tienen, and de Boer 2020), responsibilities scoring over 15 on the Multidimensional Assessment of Caring Activities (MACA‐YC18, Joseph et al. 2009), or a carer's responsibilities including at least one inappropriate responsibility or any two other caring tasks (Nagl‐Cupal et al. 2014). There are therefore challenges in comparing the results of these studies, and further research into the positive and negative impacts could aim to identify the point where responsibilities become problematic.

## 5 Conclusion

This study investigated the longitudinal impacts of young carer status on mental health, with comparison between all young carers, those with substantial responsibilities, and children without caring responsibilities. The results strengthen the evidence that the mental health impacts of caring can be negative for those with substantial responsibilities. Duration in the young carer role is also a key factor, and the findings contrast the negative mental health of long‐term carers with short‐term impacts that were often marginal or positive. Further cross‐sectional and longitudinal quantitative analysis of current data is needed to better understand young carers relative to non‐young carers, but also how impacts vary within the young carer spectrum.

## Ethics Statement

The Caring Lives study was conducted according to the guidelines of the Declaration of Helsinki, and approved by Cardiff University's School of Social Sciences Research Ethics Committee (Ref: 2869; approved 23/04/2020).

## Conflicts of Interest

The authors declare no conflicts of interest.

## Supporting information

Supporting information.

Supporting information.

## Data Availability

The Longitudinal Study of Young People in England (Next Steps) cohort study datasets are available on the UKDA website. DOI: 10.5255/UKDA‐SN‐5545‐7.
