# Supplementary material for: A Longitudinal Analysis Comparing the Mental Health of Children By Level of Young Carer Status
Source: J Adolesc. 2024 Dec 16;97(3):713–31. doi: 10.1002/jad.12448 (PMC11973838; doi:10.1002/jad.12448)
Supplement: Supplementary file 1 — Supporting information. [file JAD-97-713-s001.docx]

**Supplementary information**

Mplus SEM script: Standard young carer model

SUMMARY OF ANALYSIS

Number of groups 1

Number of observations 15923

Number of dependent variables 19

Number of independent variables 2

Number of continuous latent variables 2

Observed dependent variables

Continuous

W2SLEEPLOS W2UNDERSTR W2DIFFICUL W2ENJOYACT W2FACEPROB W2DEPRESSE

W2LOWCONFI W2HAPPY W4SLEEPLOS W4UNDERSTR W4DIFFICUL W4ENJOYACT

W4FACEPROB W4DEPRESSE W4LOWCONFI W4HAPPY

Binary and ordered categorical (ordinal)

W1CARESTAT W2CARESTAT W3CARESTAT

Observed independent variables

SEXMERGE ETHNICME

Continuous latent variables

MH2 MH4

Estimator MLR

Information matrix OBSERVED

Optimization Specifications for the Quasi-Newton Algorithm for

Continuous Outcomes

Maximum number of iterations 100

Convergence criterion 0.100D-05

Optimization Specifications for the EM Algorithm

Maximum number of iterations 500

Convergence criteria

Loglikelihood change 0.100D-02

Relative loglikelihood change 0.100D-05

Derivative 0.100D-02

Optimization Specifications for the M step of the EM Algorithm for

Categorical Latent variables

Number of M step iterations 1

M step convergence criterion 0.100D-02

Basis for M step termination ITERATION

Optimization Specifications for the M step of the EM Algorithm for

Censored, Binary or Ordered Categorical (Ordinal), Unordered

Categorical (Nominal) and Count Outcomes

Number of M step iterations 1

M step convergence criterion 0.100D-02

Basis for M step termination ITERATION

Maximum value for logit thresholds 15

Minimum value for logit thresholds -15

Minimum expected cell size for chi-square 0.100D-01

Maximum number of iterations for H1 2000

Convergence criterion for H1 0.100D-03

Optimization algorithm EMA

Integration Specifications

Type MONTECARLO

Number of integration points 750

Dimensions of numerical integration 3

Adaptive quadrature ON

Monte Carlo integration seed 0

Link LOGIT

Cholesky OFF

Input data file(s)

G:\modelvariables.dta.dat

Input data format FREE

SUMMARY OF DATA

Number of missing data patterns 689

Number of y missing data patterns 550

Number of u missing data patterns 8

COVARIANCE COVERAGE OF DATA

Minimum covariance coverage value 0.100

PROPORTION OF DATA PRESENT

Covariance Coverage

W1CAREST W2CAREST W3CAREST W2SLEEPL W2UNDERS

________ ________ ________ ________ ________

W1CAREST 0.968

W2CAREST 0.823 0.834

W3CAREST 0.762 0.761 0.771

W2SLEEPL 0.789 0.797 0.730 0.799

W2UNDERS 0.761 0.769 0.705 0.755 0.770

W2DIFFIC 0.758 0.766 0.704 0.752 0.741

W2ENJOYA 0.790 0.799 0.733 0.779 0.759

W2FACEPR 0.787 0.795 0.729 0.776 0.754

W2DEPRES 0.782 0.790 0.724 0.773 0.753

W2LOWCON 0.787 0.795 0.729 0.777 0.756

W2HAPPY 0.765 0.774 0.710 0.758 0.740

W4SLEEPL 0.686 0.686 0.687 0.659 0.638

W4UNDERS 0.683 0.683 0.684 0.658 0.637

W4DIFFIC 0.682 0.682 0.684 0.657 0.636

W4ENJOYA 0.689 0.689 0.690 0.663 0.641

W4FACEPR 0.687 0.687 0.688 0.661 0.640

W4DEPRES 0.683 0.683 0.684 0.657 0.636

W4LOWCON 0.686 0.686 0.687 0.660 0.639

W4HAPPY 0.683 0.683 0.684 0.657 0.637

SEXMERGE 0.968 0.834 0.771 0.799 0.770

ETHNICME 0.968 0.834 0.771 0.799 0.770

Covariance Coverage

W2DIFFIC W2ENJOYA W2FACEPR W2DEPRES W2LOWCON

________ ________ ________ ________ ________

W2DIFFIC 0.768

W2ENJOYA 0.757 0.800

W2FACEPR 0.755 0.784 0.797

W2DEPRES 0.751 0.777 0.774 0.792

W2LOWCON 0.756 0.780 0.779 0.780 0.797

W2HAPPY 0.739 0.764 0.760 0.756 0.762

W4SLEEPL 0.637 0.662 0.659 0.655 0.659

W4UNDERS 0.636 0.661 0.657 0.654 0.658

W4DIFFIC 0.634 0.660 0.657 0.653 0.657

W4ENJOYA 0.640 0.666 0.662 0.659 0.662

W4FACEPR 0.638 0.664 0.661 0.657 0.661

W4DEPRES 0.634 0.660 0.657 0.654 0.657

W4LOWCON 0.637 0.662 0.659 0.656 0.660

W4HAPPY 0.635 0.660 0.657 0.654 0.657

SEXMERGE 0.768 0.800 0.797 0.792 0.797

ETHNICME 0.768 0.800 0.797 0.792 0.797

Covariance Coverage

W2HAPPY W4SLEEPL W4UNDERS W4DIFFIC W4ENJOYA

________ ________ ________ ________ ________

W2HAPPY 0.775

W4SLEEPL 0.643 0.713

W4UNDERS 0.641 0.705 0.711

W4DIFFIC 0.640 0.704 0.703 0.710

W4ENJOYA 0.646 0.710 0.708 0.708 0.717

W4FACEPR 0.644 0.709 0.707 0.706 0.713

W4DEPRES 0.640 0.705 0.703 0.702 0.708

W4LOWCON 0.643 0.707 0.705 0.705 0.711

W4HAPPY 0.642 0.704 0.702 0.702 0.708

SEXMERGE 0.775 0.713 0.711 0.710 0.717

ETHNICME 0.775 0.713 0.711 0.710 0.717

Covariance Coverage

W4FACEPR W4DEPRES W4LOWCON W4HAPPY SEXMERGE

________ ________ ________ ________ ________

W4FACEPR 0.715

W4DEPRES 0.707 0.710

W4LOWCON 0.710 0.707 0.714

W4HAPPY 0.707 0.702 0.705 0.710

SEXMERGE 0.715 0.710 0.714 0.710 1.000

ETHNICME 0.715 0.710 0.714 0.710 1.000

Covariance Coverage

ETHNICME

________

ETHNICME 1.000

PROPORTION OF DATA PRESENT FOR U

Covariance Coverage

W1CAREST W2CAREST W3CAREST

________ ________ ________

W1CAREST 0.968

W2CAREST 0.823 0.834

W3CAREST 0.762 0.761 0.771

PROPORTION OF DATA PRESENT FOR Y

Covariance Coverage

W2SLEEPL W2UNDERS W2DIFFIC W2ENJOYA W2FACEPR

________ ________ ________ ________ ________

W2SLEEPL 0.799

W2UNDERS 0.755 0.770

W2DIFFIC 0.752 0.741 0.768

W2ENJOYA 0.779 0.759 0.757 0.800

W2FACEPR 0.776 0.754 0.755 0.784 0.797

W2DEPRES 0.773 0.753 0.751 0.777 0.774

W2LOWCON 0.777 0.756 0.756 0.780 0.779

W2HAPPY 0.758 0.740 0.739 0.764 0.760

W4SLEEPL 0.659 0.638 0.637 0.662 0.659

W4UNDERS 0.658 0.637 0.636 0.661 0.657

W4DIFFIC 0.657 0.636 0.634 0.660 0.657

W4ENJOYA 0.663 0.641 0.640 0.666 0.662

W4FACEPR 0.661 0.640 0.638 0.664 0.661

W4DEPRES 0.657 0.636 0.634 0.660 0.657

W4LOWCON 0.660 0.639 0.637 0.662 0.659

W4HAPPY 0.657 0.637 0.635 0.660 0.657

SEXMERGE 0.799 0.770 0.768 0.800 0.797

ETHNICME 0.799 0.770 0.768 0.800 0.797

Covariance Coverage

W2DEPRES W2LOWCON W2HAPPY W4SLEEPL W4UNDERS

________ ________ ________ ________ ________

W2DEPRES 0.792

W2LOWCON 0.780 0.797

W2HAPPY 0.756 0.762 0.775

W4SLEEPL 0.655 0.659 0.643 0.713

W4UNDERS 0.654 0.658 0.641 0.705 0.711

W4DIFFIC 0.653 0.657 0.640 0.704 0.703

W4ENJOYA 0.659 0.662 0.646 0.710 0.708

W4FACEPR 0.657 0.661 0.644 0.709 0.707

W4DEPRES 0.654 0.657 0.640 0.705 0.703

W4LOWCON 0.656 0.660 0.643 0.707 0.705

W4HAPPY 0.654 0.657 0.642 0.704 0.702

SEXMERGE 0.792 0.797 0.775 0.713 0.711

ETHNICME 0.792 0.797 0.775 0.713 0.711

Covariance Coverage

W4DIFFIC W4ENJOYA W4FACEPR W4DEPRES W4LOWCON

________ ________ ________ ________ ________

W4DIFFIC 0.710

W4ENJOYA 0.708 0.717

W4FACEPR 0.706 0.713 0.715

W4DEPRES 0.702 0.708 0.707 0.710

W4LOWCON 0.705 0.711 0.710 0.707 0.714

W4HAPPY 0.702 0.708 0.707 0.702 0.705

SEXMERGE 0.710 0.717 0.715 0.710 0.714

ETHNICME 0.710 0.717 0.715 0.710 0.714

Covariance Coverage

W4HAPPY SEXMERGE ETHNICME

________ ________ ________

W4HAPPY 0.710

SEXMERGE 0.710 1.000

ETHNICME 0.710 1.000 1.000

UNIVARIATE PROPORTIONS AND COUNTS FOR CATEGORICAL VARIABLES

W1CAREST

Category 1 0.949 14618.000

Category 2 0.051 791.000

W2CAREST

Category 1 0.942 12508.000

Category 2 0.058 764.000

W3CAREST

Category 1 0.938 11512.000

Category 2 0.062 762.000

UNIVARIATE SAMPLE STATISTICS

UNIVARIATE HIGHER-ORDER MOMENT DESCRIPTIVE STATISTICS

Variable/ Mean/ Skewness/ Minimum/ % with Percentiles

Sample Size Variance Kurtosis Maximum Min/Max 20%/60% 40%/80% Median

W2SLEEPLOS 1.773 0.954 1.000 47.54% 1.000 1.000 2.000

12718.000 0.786 0.048 4.000 5.74% 2.000 2.000

W2UNDERSTR 1.965 0.663 1.000 37.96% 1.000 2.000 2.000

12262.000 0.890 -0.525 4.000 8.31% 2.000 3.000

W2DIFFICUL 1.850 0.871 1.000 41.61% 1.000 1.000 2.000

12227.000 0.796 -0.007 4.000 6.79% 2.000 2.000

W2ENJOYACT 1.898 0.721 1.000 23.68% 1.000 2.000 2.000

12742.000 0.417 1.690 4.000 2.79% 2.000 2.000

W2FACEPROB 1.826 0.746 1.000 28.61% 1.000 2.000 2.000

12690.000 0.419 1.661 4.000 2.54% 2.000 2.000

W2DEPRESSE 1.878 0.832 1.000 44.48% 1.000 1.000 2.000

12612.000 0.927 -0.362 4.000 8.71% 2.000 3.000

W2LOWCONFI 1.704 1.137 1.000 54.07% 1.000 1.000 1.000

12693.000 0.829 0.308 4.000 6.53% 2.000 2.000

W2HAPPY 1.868 0.781 1.000 27.70% 1.000 2.000 2.000

12341.000 0.470 1.352 4.000 3.26% 2.000 2.000

W4SLEEPLOS 1.921 0.693 1.000 39.45% 1.000 2.000 2.000

11361.000 0.843 -0.442 4.000 6.93% 2.000 3.000

W4UNDERSTR 2.136 0.383 1.000 29.74% 1.000 2.000 2.000

11319.000 0.899 -0.824 4.000 9.33% 2.000 3.000

W4DIFFICUL 1.883 0.750 1.000 39.52% 1.000 2.000 2.000

11304.000 0.778 -0.221 4.000 5.94% 2.000 3.000

W4ENJOYACT 1.954 0.617 1.000 23.85% 1.000 2.000 2.000

11420.000 0.492 0.753 4.000 3.18% 2.000 2.000

W4FACEPROB 1.862 0.655 1.000 26.43% 1.000 2.000 2.000

11384.000 0.418 1.385 4.000 2.32% 2.000 2.000

W4DEPRESSE 1.905 0.734 1.000 44.80% 1.000 1.000 2.000

11312.000 0.962 -0.616 4.000 8.54% 2.000 3.000

W4LOWCONFI 1.690 1.120 1.000 55.56% 1.000 1.000 1.000

11362.000 0.822 0.214 4.000 5.85% 2.000 2.000

W4HAPPY 1.902 0.619 1.000 25.82% 1.000 2.000 2.000

11311.000 0.460 0.915 4.000 2.58% 2.000 2.000

SEXMERGE 0.508 -0.033 0.000 49.18% 0.000 0.000 1.000

15923.000 0.250 -1.999 1.000 50.82% 1.000 1.000

ETHNICMERGE 0.656 -0.656 0.000 34.42% 0.000 1.000 1.000

15923.000 0.226 -1.570 1.000 65.58% 1.000 1.000

THE MODEL ESTIMATION TERMINATED NORMALLY

MODEL FIT INFORMATION

Number of Free Parameters 50

Loglikelihood

H0 Value -210919.821

H0 Scaling Correction Factor 1.2381

for MLR

Information Criteria

Akaike (AIC) 421939.641

Bayesian (BIC) 422323.417

Sample-Size Adjusted BIC 422164.521

(n* = (n + 2) / 24)

MODEL RESULTS

Two-Tailed

Estimate S.E. Est./S.E. P-Value

MH2 BY

W2SLEEPLOS 1.000 0.000 999.000 999.000

W2UNDERSTR 1.092 0.012 90.390 0.000

W2DIFFICUL 1.026 0.013 78.384 0.000

W2ENJOYACT 0.567 0.011 53.066 0.000

W2FACEPROB 0.472 0.011 41.208 0.000

W2DEPRESSE 1.331 0.015 90.847 0.000

W2LOWCONFI 1.144 0.015 75.372 0.000

W2HAPPY 0.645 0.011 56.610 0.000

MH4 BY

W4SLEEPLOS 1.000 0.000 999.000 999.000

W4UNDERSTR 1.092 0.012 90.390 0.000

W4DIFFICUL 1.026 0.013 78.384 0.000

W4ENJOYACT 0.567 0.011 53.066 0.000

W4FACEPROB 0.472 0.011 41.208 0.000

W4DEPRESSE 1.331 0.015 90.847 0.000

W4LOWCONFI 1.144 0.015 75.372 0.000

W4HAPPY 0.645 0.011 56.610 0.000

MH4 ON

MH2 0.521 0.011 45.963 0.000

MH4 ON

W1CAREST 0.043 0.028 1.551 0.121

W2CAREST 0.026 0.027 0.956 0.339

W3CAREST 0.076 0.027 2.842 0.004

MH2 ON

W1CAREST -0.009 0.027 -0.334 0.738

W2CAREST 0.043 0.027 1.629 0.103

W2CAREST ON

W1CAREST 2.429 0.095 25.688 0.000

SEXMERGE -0.136 0.078 -1.731 0.083

ETHNICMERG -0.431 0.080 -5.371 0.000

W3CAREST ON

W2CAREST 2.829 0.092 30.868 0.000

SEXMERGE -0.098 0.081 -1.206 0.228

ETHNICMERG -0.231 0.085 -2.706 0.007

W1CAREST ON

SEXMERGE -0.097 0.073 -1.325 0.185

ETHNICMERG -0.533 0.074 -7.240 0.000

Intercepts

W2SLEEPLOS 1.836 0.007 269.319 0.000

W2UNDERSTR 2.038 0.007 281.448 0.000

W2DIFFICUL 1.859 0.007 282.507 0.000

W2ENJOYACT 1.921 0.005 412.788 0.000

W2FACEPROB 1.840 0.004 411.956 0.000

W2DEPRESSE 1.882 0.007 255.120 0.000

W2LOWCONFI 1.692 0.007 248.384 0.000

W2HAPPY 1.883 0.005 389.364 0.000

W4SLEEPLOS 1.836 0.007 269.319 0.000

W4UNDERSTR 2.038 0.007 281.448 0.000

W4DIFFICUL 1.859 0.007 282.507 0.000

W4ENJOYACT 1.921 0.005 412.788 0.000

W4FACEPROB 1.840 0.004 411.956 0.000

W4DEPRESSE 1.882 0.007 255.120 0.000

W4LOWCONFI 1.692 0.007 248.384 0.000

W4HAPPY 1.883 0.005 389.364 0.000

Thresholds

W1CAREST$1 2.541 0.066 38.792 0.000

W2CAREST$1 2.778 0.076 36.337 0.000

W3CAREST$1 2.965 0.083 35.528 0.000

Residual Variances

W2SLEEPLOS 0.458 0.008 56.335 0.000

W2UNDERSTR 0.481 0.009 54.888 0.000

W2DIFFICUL 0.425 0.009 47.745 0.000

W2ENJOYACT 0.319 0.005 61.703 0.000

W2FACEPROB 0.343 0.005 67.479 0.000

W2DEPRESSE 0.324 0.007 43.244 0.000

W2LOWCONFI 0.366 0.008 48.140 0.000

W2HAPPY 0.333 0.005 60.824 0.000

W4SLEEPLOS 0.495 0.009 56.697 0.000

W4UNDERSTR 0.505 0.008 59.706 0.000

W4DIFFICUL 0.429 0.008 52.037 0.000

W4ENJOYACT 0.370 0.006 64.443 0.000

W4FACEPROB 0.342 0.005 66.855 0.000

W4DEPRESSE 0.347 0.007 46.457 0.000

W4LOWCONFI 0.388 0.008 51.574 0.000

W4HAPPY 0.316 0.005 61.727 0.000

MH2 0.344 0.008 42.371 0.000

MH4 0.251 0.007 38.325 0.000

LOGISTIC REGRESSION ODDS RATIO RESULTS

(Est. - 1) Two-Tailed

Estimate S.E. / S.E. P-Value

W2CAREST ON

W1CAREST 11.348 1.073 9.643 0.000

SEXMERGE 0.873 0.068 -1.854 0.064

ETHNICMERG 0.650 0.052 -6.713 0.000

W3CAREST ON

W2CAREST 16.924 1.551 10.268 0.000

SEXMERGE 0.907 0.073 -1.266 0.205

ETHNICMERG 0.794 0.068 -3.044 0.002

W1CAREST ON

SEXMERGE 0.907 0.067 -1.391 0.164

ETHNICMERG 0.587 0.043 -9.562 0.000

QUALITY OF NUMERICAL RESULTS

Condition Number for the Information Matrix 0.223E-04

(ratio of smallest to largest eigenvalue)

DIAGRAM INFORMATION

Use View Diagram under the Diagram menu in the Mplus Editor to view the diagram.

If running Mplus from the Mplus Diagrammer, the diagram opens automatically.

Diagram output

d:\standardmodel.dgm

Mplus SEM script: Higher-level young carer model script

SUMMARY OF ANALYSIS

Number of groups 1

Number of observations 15906

Number of dependent variables 19

Number of independent variables 2

Number of continuous latent variables 2

Observed dependent variables

Continuous

W2SLEEPLOS W2UNDERSTR W2DIFFICUL W2ENJOYACT W2FACEPROB W2DEPRESSE

W2LOWCONFI W2HAPPY W4SLEEPLOS W4UNDERSTR W4DIFFICUL W4ENJOYACT

W4FACEPROB W4DEPRESSE W4LOWCONFI W4HAPPY

Binary and ordered categorical (ordinal)

W1CAREHO W2CAREHO W3CAREHO

Observed independent variables

SEXMERGE ETHNICME

Continuous latent variables

MH2 MH4

Estimator MLR

Information matrix OBSERVED

Optimization Specifications for the Quasi-Newton Algorithm for

Continuous Outcomes

Maximum number of iterations 100

Convergence criterion 0.100D-05

Optimization Specifications for the EM Algorithm

Maximum number of iterations 500

Convergence criteria

Loglikelihood change 0.100D-02

Relative loglikelihood change 0.100D-05

Derivative 0.100D-02

Optimization Specifications for the M step of the EM Algorithm for

Categorical Latent variables

Number of M step iterations 1

M step convergence criterion 0.100D-02

Basis for M step termination ITERATION

Optimization Specifications for the M step of the EM Algorithm for

Censored, Binary or Ordered Categorical (Ordinal), Unordered

Categorical (Nominal) and Count Outcomes

Number of M step iterations 1

M step convergence criterion 0.100D-02

Basis for M step termination ITERATION

Maximum value for logit thresholds 15

Minimum value for logit thresholds -15

Minimum expected cell size for chi-square 0.100D-01

Maximum number of iterations for H1 2000

Convergence criterion for H1 0.100D-03

Optimization algorithm EMA

Integration Specifications

Type MONTECARLO

Number of integration points 750

Dimensions of numerical integration 3

Adaptive quadrature ON

Monte Carlo integration seed 0

Link LOGIT

Cholesky OFF

Input data file(s)

G:\modelvariableshigh.dta.dat

Input data format FREE

SUMMARY OF DATA

Number of missing data patterns 713

Number of y missing data patterns 550

Number of u missing data patterns 8

COVARIANCE COVERAGE OF DATA

Minimum covariance coverage value 0.100

PROPORTION OF DATA PRESENT

Covariance Coverage

W1CAREHO W2CAREHO W3CAREHO W2SLEEPL W2UNDERS

________ ________ ________ ________ ________

W1CAREHO 0.965

W2CAREHO 0.817 0.831

W3CAREHO 0.757 0.756 0.769

W2SLEEPL 0.787 0.794 0.729 0.800

W2UNDERS 0.759 0.766 0.703 0.756 0.771

W2DIFFIC 0.757 0.764 0.702 0.753 0.742

W2ENJOYA 0.788 0.796 0.731 0.780 0.759

W2FACEPR 0.785 0.793 0.728 0.777 0.755

W2DEPRES 0.780 0.788 0.723 0.774 0.754

W2LOWCON 0.785 0.793 0.728 0.778 0.756

W2HAPPY 0.764 0.771 0.709 0.759 0.741

W4SLEEPL 0.684 0.683 0.685 0.660 0.639

W4UNDERS 0.681 0.681 0.683 0.659 0.638

W4DIFFIC 0.681 0.680 0.682 0.657 0.637

W4ENJOYA 0.687 0.687 0.689 0.663 0.642

W4FACEPR 0.685 0.684 0.687 0.662 0.641

W4DEPRES 0.681 0.680 0.682 0.658 0.637

W4LOWCON 0.684 0.683 0.685 0.661 0.640

W4HAPPY 0.681 0.681 0.682 0.658 0.637

SEXMERGE 0.965 0.831 0.769 0.800 0.771

ETHNICME 0.965 0.831 0.769 0.800 0.771

Covariance Coverage

W2DIFFIC W2ENJOYA W2FACEPR W2DEPRES W2LOWCON

________ ________ ________ ________ ________

W2DIFFIC 0.769

W2ENJOYA 0.758 0.801

W2FACEPR 0.756 0.784 0.798

W2DEPRES 0.752 0.778 0.775 0.793

W2LOWCON 0.756 0.781 0.780 0.781 0.798

W2HAPPY 0.740 0.765 0.761 0.757 0.763

W4SLEEPL 0.637 0.663 0.660 0.656 0.660

W4UNDERS 0.637 0.662 0.658 0.655 0.658

W4DIFFIC 0.635 0.660 0.657 0.654 0.657

W4ENJOYA 0.640 0.666 0.663 0.659 0.663

W4FACEPR 0.639 0.664 0.661 0.657 0.661

W4DEPRES 0.635 0.660 0.657 0.654 0.658

W4LOWCON 0.638 0.663 0.660 0.657 0.660

W4HAPPY 0.636 0.661 0.658 0.654 0.658

SEXMERGE 0.769 0.801 0.798 0.793 0.798

ETHNICME 0.769 0.801 0.798 0.793 0.798

Covariance Coverage

W2HAPPY W4SLEEPL W4UNDERS W4DIFFIC W4ENJOYA

________ ________ ________ ________ ________

W2HAPPY 0.776

W4SLEEPL 0.643 0.714

W4UNDERS 0.642 0.706 0.712

W4DIFFIC 0.641 0.705 0.704 0.711

W4ENJOYA 0.647 0.711 0.709 0.709 0.718

W4FACEPR 0.645 0.710 0.708 0.707 0.713

W4DEPRES 0.641 0.705 0.704 0.703 0.709

W4LOWCON 0.644 0.708 0.706 0.706 0.712

W4HAPPY 0.642 0.705 0.703 0.703 0.709

SEXMERGE 0.776 0.714 0.712 0.711 0.718

ETHNICME 0.776 0.714 0.712 0.711 0.718

Covariance Coverage

W4FACEPR W4DEPRES W4LOWCON W4HAPPY SEXMERGE

________ ________ ________ ________ ________

W4FACEPR 0.716

W4DEPRES 0.707 0.711

W4LOWCON 0.710 0.708 0.714

W4HAPPY 0.707 0.703 0.706 0.711

SEXMERGE 0.716 0.711 0.714 0.711 1.000

ETHNICME 0.716 0.711 0.714 0.711 1.000

Covariance Coverage

ETHNICME

________

ETHNICME 1.000

PROPORTION OF DATA PRESENT FOR U

Covariance Coverage

W1CAREHO W2CAREHO W3CAREHO

________ ________ ________

W1CAREHO 0.965

W2CAREHO 0.817 0.831

W3CAREHO 0.757 0.756 0.769

PROPORTION OF DATA PRESENT FOR Y

Covariance Coverage

W2SLEEPL W2UNDERS W2DIFFIC W2ENJOYA W2FACEPR

________ ________ ________ ________ ________

W2SLEEPL 0.800

W2UNDERS 0.756 0.771

W2DIFFIC 0.753 0.742 0.769

W2ENJOYA 0.780 0.759 0.758 0.801

W2FACEPR 0.777 0.755 0.756 0.784 0.798

W2DEPRES 0.774 0.754 0.752 0.778 0.775

W2LOWCON 0.778 0.756 0.756 0.781 0.780

W2HAPPY 0.759 0.741 0.740 0.765 0.761

W4SLEEPL 0.660 0.639 0.637 0.663 0.660

W4UNDERS 0.659 0.638 0.637 0.662 0.658

W4DIFFIC 0.657 0.637 0.635 0.660 0.657

W4ENJOYA 0.663 0.642 0.640 0.666 0.663

W4FACEPR 0.662 0.641 0.639 0.664 0.661

W4DEPRES 0.658 0.637 0.635 0.660 0.657

W4LOWCON 0.661 0.640 0.638 0.663 0.660

W4HAPPY 0.658 0.637 0.636 0.661 0.658

SEXMERGE 0.800 0.771 0.769 0.801 0.798

ETHNICME 0.800 0.771 0.769 0.801 0.798

Covariance Coverage

W2DEPRES W2LOWCON W2HAPPY W4SLEEPL W4UNDERS

________ ________ ________ ________ ________

W2DEPRES 0.793

W2LOWCON 0.781 0.798

W2HAPPY 0.757 0.763 0.776

W4SLEEPL 0.656 0.660 0.643 0.714

W4UNDERS 0.655 0.658 0.642 0.706 0.712

W4DIFFIC 0.654 0.657 0.641 0.705 0.704

W4ENJOYA 0.659 0.663 0.647 0.711 0.709

W4FACEPR 0.657 0.661 0.645 0.710 0.708

W4DEPRES 0.654 0.658 0.641 0.705 0.704

W4LOWCON 0.657 0.660 0.644 0.708 0.706

W4HAPPY 0.654 0.658 0.642 0.705 0.703

SEXMERGE 0.793 0.798 0.776 0.714 0.712

ETHNICME 0.793 0.798 0.776 0.714 0.712

Covariance Coverage

W4DIFFIC W4ENJOYA W4FACEPR W4DEPRES W4LOWCON

________ ________ ________ ________ ________

W4DIFFIC 0.711

W4ENJOYA 0.709 0.718

W4FACEPR 0.707 0.713 0.716

W4DEPRES 0.703 0.709 0.707 0.711

W4LOWCON 0.706 0.712 0.710 0.708 0.714

W4HAPPY 0.703 0.709 0.707 0.703 0.706

SEXMERGE 0.711 0.718 0.716 0.711 0.714

ETHNICME 0.711 0.718 0.716 0.711 0.714

Covariance Coverage

W4HAPPY SEXMERGE ETHNICME

________ ________ ________

W4HAPPY 0.711

SEXMERGE 0.711 1.000

ETHNICME 0.711 1.000 1.000

UNIVARIATE PROPORTIONS AND COUNTS FOR CATEGORICAL VARIABLES

W1CAREHO

Category 1 0.992 15222.000

Category 2 0.008 121.000

W2CAREHO

Category 1 0.991 13098.000

Category 2 0.009 114.000

W3CAREHO

Category 1 0.990 12108.000

Category 2 0.010 126.000

UNIVARIATE SAMPLE STATISTICS

UNIVARIATE HIGHER-ORDER MOMENT DESCRIPTIVE STATISTICS

Variable/ Mean/ Skewness/ Minimum/ % with Percentiles

Sample Size Variance Kurtosis Maximum Min/Max 20%/60% 40%/80% Median

W2SLEEPLOS 1.773 0.954 1.000 47.54% 1.000 1.000 2.000

12718.000 0.786 0.048 4.000 5.74% 2.000 2.000

W2UNDERSTR 1.965 0.663 1.000 37.96% 1.000 2.000 2.000

12262.000 0.890 -0.525 4.000 8.31% 2.000 3.000

W2DIFFICUL 1.850 0.871 1.000 41.61% 1.000 1.000 2.000

12227.000 0.796 -0.007 4.000 6.79% 2.000 2.000

W2ENJOYACT 1.898 0.721 1.000 23.68% 1.000 2.000 2.000

12742.000 0.417 1.690 4.000 2.79% 2.000 2.000

W2FACEPROB 1.826 0.746 1.000 28.61% 1.000 2.000 2.000

12690.000 0.419 1.661 4.000 2.54% 2.000 2.000

W2DEPRESSE 1.878 0.832 1.000 44.48% 1.000 1.000 2.000

12612.000 0.927 -0.362 4.000 8.71% 2.000 3.000

W2LOWCONFI 1.704 1.137 1.000 54.07% 1.000 1.000 1.000

12693.000 0.829 0.308 4.000 6.53% 2.000 2.000

W2HAPPY 1.868 0.781 1.000 27.70% 1.000 2.000 2.000

12341.000 0.470 1.352 4.000 3.26% 2.000 2.000

W4SLEEPLOS 1.921 0.693 1.000 39.45% 1.000 2.000 2.000

11361.000 0.843 -0.442 4.000 6.93% 2.000 3.000

W4UNDERSTR 2.136 0.383 1.000 29.74% 1.000 2.000 2.000

11319.000 0.899 -0.824 4.000 9.33% 2.000 3.000

W4DIFFICUL 1.883 0.750 1.000 39.52% 1.000 2.000 2.000

11304.000 0.778 -0.221 4.000 5.94% 2.000 3.000

W4ENJOYACT 1.954 0.617 1.000 23.85% 1.000 2.000 2.000

11420.000 0.492 0.753 4.000 3.18% 2.000 2.000

W4FACEPROB 1.862 0.655 1.000 26.43% 1.000 2.000 2.000

11384.000 0.418 1.385 4.000 2.32% 2.000 2.000

W4DEPRESSE 1.905 0.734 1.000 44.80% 1.000 1.000 2.000

11312.000 0.962 -0.616 4.000 8.54% 2.000 3.000

W4LOWCONFI 1.690 1.120 1.000 55.56% 1.000 1.000 1.000

11362.000 0.822 0.214 4.000 5.85% 2.000 2.000

W4HAPPY 1.902 0.619 1.000 25.82% 1.000 2.000 2.000

11311.000 0.460 0.915 4.000 2.58% 2.000 2.000

SEXMERGE 0.508 -0.034 0.000 49.16% 0.000 0.000 1.000

15906.000 0.250 -1.999 1.000 50.84% 1.000 1.000

ETHNICMERGE 0.656 -0.657 0.000 34.39% 0.000 1.000 1.000

15906.000 0.226 -1.568 1.000 65.61% 1.000 1.000

THE MODEL ESTIMATION TERMINATED NORMALLY

MODEL FIT INFORMATION

Number of Free Parameters 50

Loglikelihood

H0 Value -204711.639

H0 Scaling Correction Factor 1.2530

for MLR

Information Criteria

Akaike (AIC) 409523.279

Bayesian (BIC) 409907.001

Sample-Size Adjusted BIC 409748.105

(n* = (n + 2) / 24)

MODEL RESULTS

Two-Tailed

Estimate S.E. Est./S.E. P-Value

MH2 BY

W2SLEEPLOS 1.000 0.000 999.000 999.000

W2UNDERSTR 1.092 0.012 90.397 0.000

W2DIFFICUL 1.026 0.013 78.375 0.000

W2ENJOYACT 0.567 0.011 53.058 0.000

W2FACEPROB 0.472 0.011 41.218 0.000

W2DEPRESSE 1.331 0.015 90.844 0.000

W2LOWCONFI 1.145 0.015 75.395 0.000

W2HAPPY 0.645 0.011 56.623 0.000

MH4 BY

W4SLEEPLOS 1.000 0.000 999.000 999.000

W4UNDERSTR 1.092 0.012 90.397 0.000

W4DIFFICUL 1.026 0.013 78.375 0.000

W4ENJOYACT 0.567 0.011 53.058 0.000

W4FACEPROB 0.472 0.011 41.218 0.000

W4DEPRESSE 1.331 0.015 90.844 0.000

W4LOWCONFI 1.145 0.015 75.395 0.000

W4HAPPY 0.645 0.011 56.623 0.000

MH4 ON

MH2 0.521 0.011 46.050 0.000

MH4 ON

W1CAREHOUR 0.061 0.069 0.893 0.372

W2CAREHOUR 0.175 0.071 2.465 0.014

W3CAREHOUR 0.080 0.074 1.077 0.282

MH2 ON

W1CAREHOUR -0.017 0.065 -0.258 0.797

W2CAREHOUR -0.054 0.063 -0.862 0.389

W2CAREHOSH ON

W1CAREHOUR 3.313 0.281 11.788 0.000

SEXMERGE -0.262 0.194 -1.351 0.177

ETHNICMERG -0.770 0.195 -3.951 0.000

W3CAREHOSH ON

W2CAREHOUR 3.429 0.288 11.916 0.000

SEXMERGE -0.462 0.190 -2.435 0.015

ETHNICMERG -0.707 0.189 -3.748 0.000

W1CAREHOSH ON

SEXMERGE -0.326 0.186 -1.756 0.079

ETHNICMERG -0.863 0.183 -4.708 0.000

Intercepts

W2SLEEPLOS 1.841 0.007 274.425 0.000

W2UNDERSTR 2.043 0.007 287.942 0.000

W2DIFFICUL 1.864 0.006 288.726 0.000

W2ENJOYACT 1.923 0.005 416.141 0.000

W2FACEPROB 1.842 0.004 413.124 0.000

W2DEPRESSE 1.888 0.007 263.357 0.000

W2LOWCONFI 1.698 0.007 254.841 0.000

W2HAPPY 1.886 0.005 394.917 0.000

W4SLEEPLOS 1.841 0.007 274.425 0.000

W4UNDERSTR 2.043 0.007 287.942 0.000

W4DIFFICUL 1.864 0.006 288.726 0.000

W4ENJOYACT 1.923 0.005 416.141 0.000

W4FACEPROB 1.842 0.004 413.124 0.000

W4DEPRESSE 1.888 0.007 263.357 0.000

W4LOWCONFI 1.698 0.007 254.841 0.000

W4HAPPY 1.886 0.005 394.917 0.000

Thresholds

W1CAREHO$1 4.190 0.147 28.430 0.000

W2CAREHO$1 4.345 0.174 24.925 0.000

W3CAREHO$1 4.113 0.159 25.946 0.000

Residual Variances

W2SLEEPLOS 0.458 0.008 56.350 0.000

W2UNDERSTR 0.481 0.009 54.918 0.000

W2DIFFICUL 0.425 0.009 47.744 0.000

W2ENJOYACT 0.319 0.005 61.714 0.000

W2FACEPROB 0.343 0.005 67.481 0.000

W2DEPRESSE 0.324 0.007 43.255 0.000

W2LOWCONFI 0.366 0.008 48.139 0.000

W2HAPPY 0.333 0.005 60.812 0.000

W4SLEEPLOS 0.496 0.009 56.693 0.000

W4UNDERSTR 0.505 0.008 59.697 0.000

W4DIFFICUL 0.429 0.008 52.032 0.000

W4ENJOYACT 0.370 0.006 64.438 0.000

W4FACEPROB 0.342 0.005 66.876 0.000

W4DEPRESSE 0.348 0.007 46.422 0.000

W4LOWCONFI 0.388 0.008 51.576 0.000

W4HAPPY 0.316 0.005 61.717 0.000

MH2 0.344 0.008 42.337 0.000

MH4 0.251 0.007 38.294 0.000

LOGISTIC REGRESSION ODDS RATIO RESULTS

(Est. - 1) Two-Tailed

Estimate S.E. / S.E. P-Value

W2CAREHOSH ON

W1CAREHOUR 27.458 7.717 3.429 0.001

SEXMERGE 0.769 0.149 -1.545 0.122

ETHNICMERG 0.463 0.090 -5.951 0.000

W3CAREHOSH ON

W2CAREHOUR 30.845 8.876 3.362 0.001

SEXMERGE 0.630 0.119 -3.095 0.002

ETHNICMERG 0.493 0.093 -5.450 0.000

W1CAREHOSH ON

SEXMERGE 0.722 0.134 -2.076 0.038

ETHNICMERG 0.422 0.077 -7.473 0.000

QUALITY OF NUMERICAL RESULTS

Condition Number for the Information Matrix 0.110E-03

(ratio of smallest to largest eigenvalue)

DIAGRAM INFORMATION

Use View Diagram under the Diagram menu in the Mplus Editor to view the diagram.

If running Mplus from the Mplus Diagrammer, the diagram opens automatically.

Diagram output

d:\standardmodelhigh.dgm
