# Supplementary material for: A Longitudinal Analysis Comparing the Mental Health of Children By Level of Young Carer Status
Source: J Adolesc. 2024 Dec 16;97(3):713–31. doi: 10.1002/jad.12448 (PMC11973838; doi:10.1002/jad.12448)
Supplement: Supplementary file 2 — Supporting information. [file JAD-97-713-s002.docx]

**Supplementary information**

*PCA and CFA results*

The Principal Component Analysis (PCA) utilised multiple tools to assess the optimal number of factors for each wave of mental health data. The Velicer MAP (*W2*=0.037; *W4*=0.032), VSS Complexity 1 (*W2*=0.82; *W4*=0.82), and the scree plots (Tables 4 and 5, and Figure 4) indicate the need for a single mental health factor.

| PCA results (VSS1, VSS2, Velicer) for Wave 2 data. |
| --- |
| 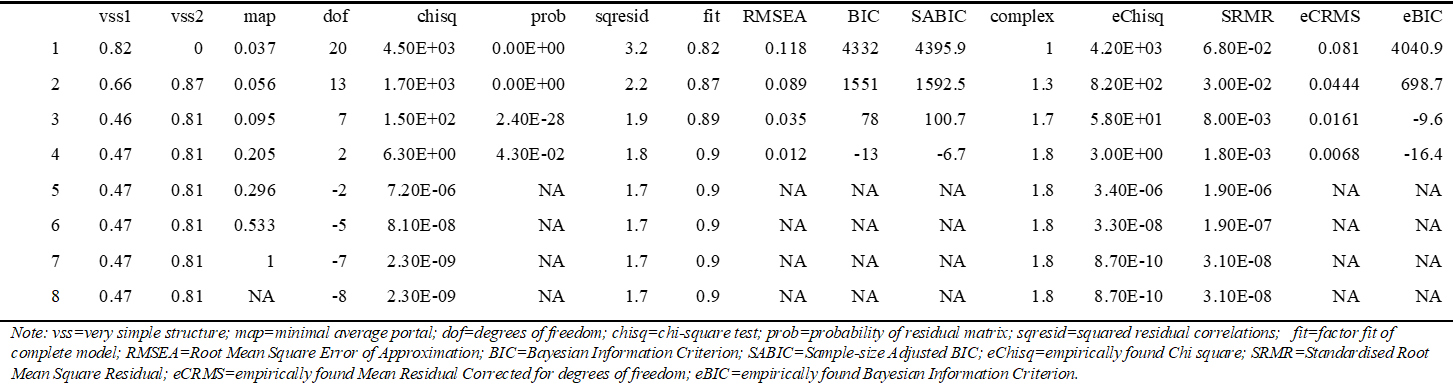 |

| PCA results (VSS1, VSS2, Velicer) for Wave 4 data. |
| --- |
| 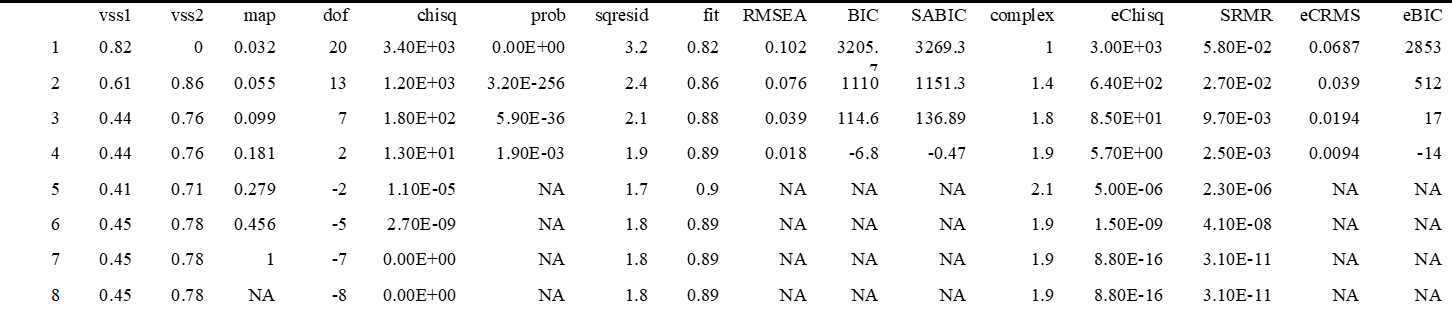 |

| Scree plots for Wave Two (left) and Wave Four data. |
| --- |
| 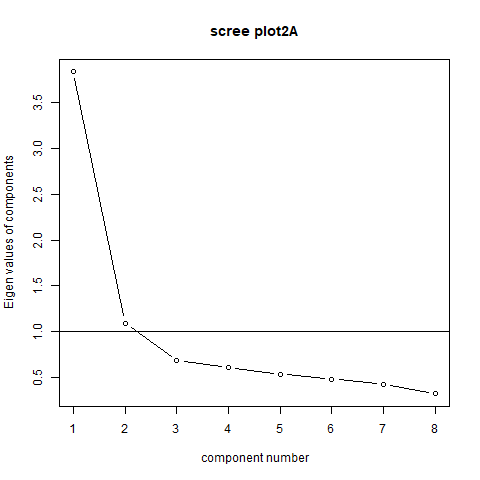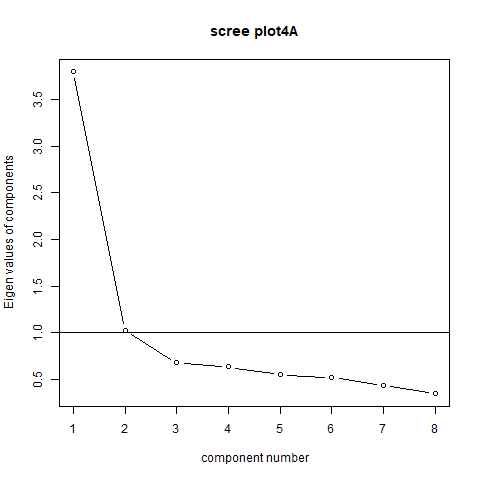 **W2**  **W4** |

A Maximum Likelihood confirmatory factor analysis indicated medium to strong relationships between the individual mental health indicators and the factors (Table 6), from 0.42 (*W2FaceProblems*) to 0.81 (*W2Depressed*). Communality, the level of variance of each indicator with the other variables, was low for facing problems (*W2FaceProblems* = 0.18; *W4FaceProblems* = 0.19), but otherwise ranged from moderate (*W2EnjoyActivities* = 0.23) to high (*W2Depressed* = 0.66).

| Maximum likelihood confirmatory factor analysis results for Wave Two (left) and Four data. | | | | | | | | | | |
| --- | --- | --- | --- | --- | --- | --- | --- | --- | --- | --- |
|  | ML1 | h2 | u2 | com |  |  | ML1 | h2 | u2 | com |
| W2SleepLoss | 0.64 | 0.42 | 0.58 | 1 |  | W4SleepLoss | 0.64 | 0.41 | 0.59 | 1 |
| W2UnderStrain | 0.68 | 0.46 | 0.54 | 1 |  | W4UnderStrain | 0.66 | 0.44 | 0.56 | 1 |
| W2Difficulties | 0.69 | 0.47 | 0.53 | 1 |  | W4Difficulties | 0.67 | 0.44 | 0.56 | 1 |
| W2EnjoyActivities | 0.48 | 0.23 | 0.77 | 1 |  | W4EnjoyActivities | 0.51 | 0.26 | 0.74 | 1 |
| W2FaceProblems | 0.42 | 0.18 | 0.82 | 1 |  | W4FaceProblems | 0.43 | 0.19 | 0.81 | 1 |
| W2Depressed | 0.81 | 0.66 | 0.34 | 1 |  | W4Depressed | 0.8 | 0.64 | 0.36 | 1 |
| W2LowConfidence | 0.75 | 0.57 | 0.43 | 1 |  | W4LowConfidence | 0.72 | 0.53 | 0.47 | 1 |
| W2Happy | 0.54 | 0.29 | 0.71 | 1 |  | W4Happy | 0.57 | 0.32 | 0.68 | 1 |
|  |  |  |  |  |  |  |  |  |  |  |
|  |  | ML1 |  |  |  |  | | ML1 |  |  |
| SS loadings | | 3.28 |  |  |  | SS loadings | | 3.23 |  |  |
| Proportion Var | | 0.41 |  |  |  | Proportion Var | | 0.4 |  |  |
